# Wnt Pathway: An Emerging Player in Vascular and Traumatic Mediated Brain Injuries

**DOI:** 10.3389/fphys.2020.565667

**Published:** 2020-09-18

**Authors:** Romain Menet, Sarah Lecordier, Ayman ElAli

**Affiliations:** ^1^Neuroscience Axis, Research Center of CHU de Québec – Université Laval, Quebec City, QC, Canada; ^2^Department of Psychiatry and Neuroscience, Faculty of Medicine, Université Laval, Quebec City, QC, Canada

**Keywords:** Wnt pathway, cerebrovascular diseases, traumatic brain injury, signal transduction, neurovascular interactions

## Abstract

The Wnt pathway, which comprises the canonical and non-canonical pathways, is an evolutionarily conserved mechanism that regulates crucial biological aspects throughout the development and adulthood. Emergence and patterning of the nervous and vascular systems are intimately coordinated, a process in which Wnt pathway plays particularly important roles. In the brain, Wnt ligands activate a cell-specific surface receptor complex to induce intracellular signaling cascades regulating neurogenesis, synaptogenesis, neuronal plasticity, synaptic plasticity, angiogenesis, vascular stabilization, and inflammation. The Wnt pathway is tightly regulated in the adult brain to maintain neurovascular functions. Historically, research in neuroscience has emphasized essentially on investigating the pathway in neurodegenerative disorders. Nonetheless, emerging findings have demonstrated that the pathway is deregulated in vascular- and traumatic-mediated brain injuries. These findings are suggesting that the pathway constitutes a promising target for the development of novel therapeutic protective and restorative interventions. Yet, targeting a complex multifunctional signal transduction pathway remains a major challenge. The review aims to summarize the current knowledge regarding the implication of Wnt pathway in the pathobiology of ischemic and hemorrhagic stroke, as well as traumatic brain injury (TBI). Furthermore, the review will present the strategies used so far to manipulate the pathway for therapeutic purposes as to highlight potential future directions.

## Introduction

The Wnt pathway regroups evolutionarily conserved intracellular signal transduction cascades that regulate key biological aspects, such as cell proliferation, polarity, migration, and fate determination during development (Willert and Nusse, 2012). Wnt proteins have been discovered 30 years ago (Nusse and Varmus, 1982), and the name is an abbreviation resulting from the fusion of the name of Drosophila segment polarity gene *“Wingless”* and that of its vertebrate proto-oncogene orthologous gene *“Integrated, Int-1”* (Nusse and Varmus, 1982). Nineteen Wnt genes have been identified so far in humans and rodents, clustering into 12 subfamilies (Foulquier et al., 2018). Wnts are secreted glycoproteins that activate intracellular signals upon binding to cell-specific transmembrane receptors (Hermann and ElAli, 2012). Wnt proteins activate different pathways that comprise the canonical pathway, which depends upon β-catenin-mediated gene regulation, and the non-canonical pathway that includes the planar cell polarity (PCP) and calcium (Ca^2+^) pathways, which are both β-catenin-independent (Croce and McClay, 2008). The canonical Wnt pathway is mediated essentially by the action of Wnt1, Wnt3, Wnt3a, Wnt7a, Wnt7b, Wnt8 and Wnt9 ligands, and involves the recruitment of low-density lipoprotein receptor-related protein-5/6 (LRP5/6) to Frizzled (Fzd) receptors to form a transmembrane receptor complex, and the non-canonical Wnt pathway is mediated essentially by Wnt4, Wnt5a, Wnt6, and Wnt11 ligands, without the involvement of LRP5/6 (Croce and McClay, 2008). In the brain, Wnt proteins are secreted essentially by neurons and astrocytes, and act as cell-specific ligands that orchestrate a wide range of biological processes during development and adulthood (Hermann and ElAli, 2012; Noelanders and Vleminckx, 2017). In the developing brain, Wnt pathway has been shown to regulate neural patterning, neurogenesis, axon guidance, synaptogenesis, and vascular development (Hermann and ElAli, 2012; Lambert et al., 2016; Noelanders and Vleminckx, 2017; Jaworski et al., 2019). Recent evidence is demonstrating that Wnt pathway is required to maintain brain homeostasis and functioning during lifespan by fine-tuning adult neurogenesis, synaptic plasticity, vascular stability, blood-brain barrier (BBB) integrity, and inflammation (Hermann and ElAli, 2012; Noelanders and Vleminckx, 2017). The emerging findings are indicating that the Wnt pathway is deregulated in several brain disorders, namely Alzheimer’s disease (AD), anticipating its importance as a novel target for the development of new therapies (Hermann and ElAli, 2012). Dysfunction of the Wnt pathway in neurodegenerative disorders is largely covered in the literature. In the recent years, deregulation of the Wnt pathway has been reported in vascular- and traumatic-mediated brain injuries, outlining a direct and major impact on the mechanisms related to injury, as well as protection and regeneration. Yet, there is still a gap in the literature in this particular field. In this review, we will summarize the implication of Wnt pathway in the pathobiology of ischemic and hemorrhagic stroke, as well as traumatic brain injury (TBI), and outline its role in the development of novel therapeutic interventions for these neurological conditions.

## The Canonical Wnt Pathway

In the canonical Wnt pathway, Wnt ligands bind to 10 different Fzd receptors (Wang et al., 2006). The interaction between Wnt and Fzd receptors requires LRP5/6, which acts as a co-receptor. The complex Wnt-Fzd-LRP5/6 recruits and activates the scaffold protein Disheveled (Dvl), which induces the disassembly of β-catenin destruction complex, which comprises the adenomatous polyposis coli (APC), Axin, casein kinase-1α (CK1α), and glycogen synthase kinase3-β (GSK3β) (Croce and McClay, 2008). LRP5/6 phosphorylation by CK1α induces inhibition of the destruction complex (Croce and McClay, 2008). In the presence of Wnt ligands, the serine kinase activity of GSK3β is inhibited, resulting in the disassembly of the destruction complex, leading to the stabilization and accumulation of β-catenin in the cytosol and subsequent translocation into the nucleus (Hermann and ElAli, 2012). In the nucleus, β-catenin binds and activates the lymphoid enhancer factor (LEF)/T cell factor (TCF) transcription factor to regulate the expression of Wnt target genes (Niehrs, 2012). TCF/LEF activation by β-catenin regulates several genes implicated in neurogenesis, synaptogenesis, neuronal plasticity, synaptic plasticity, BBB formation, cell survival, and inflammation (Liebner et al., 2008; Hur and Zhou, 2010; Marchetti and Pluchino, 2013; Zhou Y. et al., 2014; Ma and Hottiger, 2016). The interaction of Wnt with Fzd-LRP5/6 receptor complex is tightly regulated to assure an adequate activation of the pathway (He et al., 2016; Lambert et al., 2016). The endogenous secreted proteins Dickkopf-1 (Dkk1) and sclerostin (Scl) play particular important roles in modulating pathway activation (He et al., 2016; Lambert et al., 2016).

LRP5 and LRP6 are 70% identical and represent a unique group of the LDLR family (He et al., 2004; MacDonald and He, 2012). These single transmembrane receptors have an extracellular domain containing 4-tandem β-propeller (E1–E4) (MacDonald and He, 2012). Some Wnt ligands such as Wnt1, Wnt2, Wnt2b, Wnt6, Wnt8a, Wnt9a and Wnt9b interact with E1, whereas others such as Wnt3 and Wnt3a prefer E3 and E4 (Takanari et al., 1990; Bourhis et al., 2010; Rochefort, 2014). The domains that are implicated in binding other Wnt ligands such as Wnt7a and Wnt7b remain unknown (Takanari et al., 1990). It has been shown that Dkk1 binds to all β-propeller domains of LRP5 and LRP6, thus inhibiting Wnt1, Wnt9b, and Wnt3a for binding to LRP5/6 and consequently preventing pathway activation (Bourhis et al., 2010; Rochefort, 2014). Furthermore, Dkk1 binds to E3 and E4 fragments, thus competing with Wnt3a for binding to LRP6, and preventing the formation of the Wnt3a-Fzd8-LRP6 complex. Inhibition of Wnt3a binding to LRP6 by Dkk1 does not disrupt Wnt3a-Fzd8 interaction, but rather prevents the formation of Wnt-Fzd-LRP5/6 complex (Bourhis et al., 2010; Rochefort, 2014). Scl binds the first β-propeller of LRP5 and LRP6 to inhibit the biological activity of Wnt1 (Rochefort, 2014), and Wnt9b (Bourhis et al., 2010), respectively, and similar to Dkk1, prevents pathway activation. In addition, Dkk1 and Scl can use co-receptors, such as Kremen-1/2, to increase their inhibitory activity by facilitating the internalization of the Fzd-LRP5/6 receptor complex (Mao et al., 2002). In the healthy brain, Wnt pathway basal activity is required to maintain tissue homeostasis. However, accumulating evidence is suggesting that the pathway is deregulated in brain injuries associated to cerebrovascular diseases and trauma, impacting adult neurogenesis, neuronal plasticity, synaptic plasticity, angiogenesis, vascular stability and the immune response (He et al., 2016; Lambert et al., 2016).

## The Non-Canonical Wnt Pathway

The non-canonical Wnt pathway comprises Wnt/PCP and Wnt/Ca^2+^ pathways, which are β-catenin-independent, and are largely associated to cell mobility and differentiation (Ng et al., 2019). The Wnt/PCP pathway is initiated when Fzd receptors activate a cascade of intracellular cascades involving the small guanosine triphosphate (GTP)ases, Ras-related C3 botulinum toxin substrate-1 (RAC1), and Ras homolog gene family member A (RHOA), cell division cycle 42 (CDC42), as well as the c-Jun N-terminal-kinase (JNK), independently of LRP5/6 (Habas and Dawid, 2005). The Wnt/PCP pathway is divided into two sub-branches: the RHOA and its effector Rho-associated protein kinase-1/2 (ROCK1/2) “RHOA/ROCK pathway” and JNK effector c-Jun “JNK/c-Jun pathway.” RHOA/ROCK pathway does not always involve the recruitment of key components of the canonical pathway including Wnt itself, but involves mostly Dvl and specifically its PDZ (post-synaptic density protein-95, PSD95/disks large homolog, DLG/zonula occludens-1, ZO1) and DEP (Dvl/EGg laying defective, EGL-10/Pleckstrin) domains, as well as the Dvl-associated activator of morphogenesis-1 (DAAM1) (Habas et al., 2001). These domains link Fzd and Dvl to the small GTPases RHO, which in turn activate their effector ROCK, leading to cytoskeletal reorganization (Veeman et al., 2003). The JNK/c-Jun pathway involves as well Dvl that interacts through its DEP domain with RAC1 to form a complex independently of DAAM1, stimulating JNK activity and mediating profilin binding to actin (Habas et al., 2001; Veeman et al., 2003). The PCP pathway regulates a variety of cellular processes including planar polarity, cell mobility, and cell migration of neural crest cells (Veeman et al., 2003; Komiya and Habas, 2008; Ng et al., 2019).

The Wnt/Ca^2+^ pathway shares several components with the PCP pathway. The pathway is activated when Wnt ligands bind to the Fzd receptors, which activates heterotrimeric G proteins, stimulating the release of Ca^2+^ from intracellular stores (Marchetti and Pluchino, 2013). The increased Ca^2+^ concentration subsequently activates various Ca^2+^-dependent effectors, namely protein kinase C (PKC), Ca^2+^-calmodulin-dependent protein kinase II (CaMKII), and the Ca^2+^-calmodulin-sensitive protein phosphatase calcineurin (Marchetti and Pluchino, 2013; Ng et al., 2019). Several downstream components of the Wnt/Ca^2+^ pathway have been shown to interact with the canonical Wnt pathway. Furthermore, the association of Fzd receptors with Knypek (Kny), RAR-related orphan receptor-2 (ROR2), or related to receptor tyrosine kinase (RYK) receptors can activate JNK, promoting target gene expression through Activator protein-1 (AP1) (Marchetti and Pluchino, 2013). The Wnt/Ca^2+^ pathway is implicated in regulating dorsal axis formation, cell adhesion, migration, and tissue separation during embryogenesis (Komiya and Habas, 2008). Little is known about the physiological role of the non-canonical Wnt pathway in the adult brain. Nonetheless, the recent findings suggest that the pathway is deregulated in brain injuries, such as cerebrovascular diseases and trauma, impacting adult neurogenesis, angiogenesis, BBB permeability and the immune response (Johnson and Nakamura, 2007).

## The Wnt Pathway in Brain Physiology

### Implication in Neurogenesis

The adult hippocampal stem/progenitor cells (AHPs) express receptors and several components for the Wnt pathway (Lie et al., 2005). It has been shown that the canonical Wnt pathway exhibits a basal activity, and Wnt3 is expressed in the hippocampal neurogenic niche (Lie et al., 2005). Importantly, Wnt3 overexpression was sufficient to increase neurogenesis from AHPs *in vitro* and *in vivo* (Lie et al., 2005), outlining the importance of the pathway in regulating this process. In addition, β-catenin was detected in progenitor cells within the adult sub-ventricular zone (SVZ) of Axin2-d2EGFP transgenic mice, a mouse line reporter for Wnt pathway activity (Lie et al., 2005). Indeed, when canonical Wnt pathway is blocked via the expression of a dominant negative LEF1 (dnLEF1) in AHPs, a reduction of neuronal differentiation was observed in hippocampal cultures (Lie et al., 2005). The presence of astrocyte-derived secreted Fzd receptor progenitor-2/3 (sFRP2/3), which acts as decoy receptor negatively regulating pathway activity, decreased the percentage of AHPs, outlining canonical Wnt pathway participation to the differentiation of AHPs through factors derived from the hippocampal astrocytes (Lie et al., 2005). Furthermore, inhibition of GSK3β promoted both the proliferation and neuronal differentiation of human neural progenitor cells (NPCs) (Esfandiari et al., 2012), and increased neurogenesis in the sub-granular zone (SGZ) of adult mice (Adachi et al., 2007). Inhibition of Wnt signaling using dominant negative Wnt (dnWnt) (Jessberger et al., 2009), and dnWnt1 (Lie et al., 2005) reduced the level of adult hippocampal neurogenesis, and impaired the cognitive functions of mice. Importantly, the retroviral injection of Dkk1 into the SVZ reduced the proliferation of mammalian achaete-scute homolog-1 (MASH1)^+^ NPCs (Adachi et al., 2007). Furthermore, in Nestin-Dkk1 mice in which Dkk1 is specifically depleted in NPCs, neurons and glial cells, an increased number of neural progenitors was observed, accompanied by an enhanced dendritic complexity and neuronal activity in the dentate gyrus (DG) (Seib et al., 2013). Interestingly, reduction of the paracrine Wnt3 during aging has been shown to impair adult neurogenesis by modulating expression of the neuronal-lineage factors NeuroD1, retrotransposon L1, and doublecortin (Dcx) (Okamoto et al., 2011). Finally, the loss of Wnt7a expression reduced the expansion of NPCs *in vitro*, and in Wnt7a^–/–^ mice a decreased number of newborn neurons at the SGZ was observed, accompanied by altered neuronal maturation translated by an impaired dendritic development, thus linking Wnt7a to self-renewal and differentiation (Qu et al., 2013).

### Implication in Synaptogenesis

Wnt ligands have been shown directly modulate the function as well as the architecture of the pre-synaptic regions (Cerpa et al., 2008). Wnt3a and Wnt7a have been reported to stimulate exocytosis and recycling of synaptic vesicles in hippocampal neurons, thus enhancing the synaptic transmission and plasticity (Cerpa et al., 2008; Rosso and Inestrosa, 2013). Wnt3a and Wnt7a, which are expressed as well by post-synaptic components, have been shown to promote the assembly of pre-synaptic structures at the early stages of synapse formation (Cerpa et al., 2008). Wnt7a stimulated the pre-synaptic assembly via inhibition of GSK3β, without transcriptional regulation (Cerpa et al., 2008), and promoted dendritic spine growth and synaptic strength via a CaMKII-dependent mechanism *in vitro* and *in vivo* (Ciani et al., 2011). Wnt7a increased the density and maturity of dendritic spines, whereas Wnt7a-Dvl1 deficient mice exhibited several defects in spine morphogenesis and mossy fiber CA3 synaptic transmission in the hippocampus (Ciani et al., 2011). Furthermore, Wnt5a induced short-term changes in the clustering of PSD95 and modulated glutamatergic synaptic transmission (Farías et al., 2009). In cultured hippocampal neurons, the tyrosine-protein kinase transmembrane receptors, receptor tyrosine kinase-like orphan receptor-1/2 (Ror1/2), have been demonstrated to play an important role in synapse formation by interacting with Wnt5a to increase synapse formation (Paganoni et al., 2010). The length and number of synapses significantly decreased when Ror1/2 were depleted (Paganoni et al., 2010). Overexpression of Fzd1 receptor increased the pre-synaptic clustering (Varela-Nallar et al., 2009). Interestingly, it has been shown that the astrocytic layers in the DG play an essential role in triggering neuronal differentiation of hippocampal neural stem cells (NSCs) (Kuwabara et al., 2009). Astrocyte-derived Wnt3 has been shown to promote NSC differentiation in a paracrine manner by increasing the expression of synapsin-I and tubulin-III (Okamoto et al., 2011). Wnt3 overexpression in aged primary astrocytes using lentivirus (LV)-expressing Wnt3 significantly improved neurogenesis (Okamoto et al., 2011). Furthermore, GSK3β overexpression has been shown to alter dendritic branching and reducing the number of functional synapses of granule cells in DG (Llorens-Martín et al., 2013). In contrast, GSK3β depletion in the cortex and hippocampus of mice potently reduced spine density associated to loss of persistent spines and destabilization of the new spines (Ochs et al., 2015). These changes were accompanied with an impaired α-amino-3-hydroxy-5-methyl-4-isoxazolepropionic acid (AMPA) receptor-dependent excitatory post-synaptic currents (Ochs et al., 2015). Finally, TAT-TI-JIP, a JNK inhibitor, prevented the increase in the number of PSD95 clusters induced by Wnt5a (Farías et al., 2009).

### Implication in Angiogenesis

The Wnt pathway has been shown to play an important role in angiogenesis. Indeed, cadherin-5 (Cdh5)(PAC)-Cre^ERT2^;Ctnnb1^floxed^, or Ctnnb1^iΔEC^ transgenic mice, in which β-catenin depletion is specifically induced in endothelial cells, showed low vessel area and reduced number of vascular branch points (Martowicz et al., 2019). In addition, Wnt7a and Wnt7b have been identified as key regulator of normal angiogenesis in the ventral region of the brain, as the combined inactivation of both ligands caused severe defects in brain angiogenesis (Engelhardt, 2003). Indeed, Wnt7a and Wnt7b depletion caused vascular malformations in mice (Daneman et al., 2009). For instance, in Wnt7a^+/–^;Wnt7b^–/–^ mice, which are deficient in Wnt7b, a thickened vascular plexus was observed, whereas in Wnt7a^–/–^;Wnt7b^–/–^ mice, which are deficient for both Wnt7a and Wnt7b, large vascular plexus dilations were observed (Daneman et al., 2009). Wnt3a also plays a major role in angiogenesis but has a moderate effect compared to the two others Wnt7 ligands. Treatment of the human endothelial cell line EAhy926 with Wnt3a up-regulated the nuclear levels of nuclear β-catenin, accompanied by an enhanced transcriptional activity of LEF1, which induced matrix metalloproteinase-2 (MMP2) expression, an effect that was completely abrogated in the presence of LEF1 siRNA (Planutiene et al., 2011). In Fzd7*^iECKO^* mice, which are deficient in Fzd7 in endothelial cells, a reduced level of the activated form of β-catenin was observed, and accompanied by a downregulation of Axin2 and LEF1 gene expression (Peghaire et al., 2016). In these mice, the spreading and the percentage of vascularization showed a strong delay in vascular formation in the retina (Peghaire et al., 2016). In Fzd4^–/–^ mice, the neuronal degeneration observed in the brain was essentially caused by vascular dysfunction, due to misshapen capillaries and protrusions of the endothelial processes into the vessel lumen (Ye et al., 2009). Interestingly, the canonical Wnt pathway has been shown to suppress early sphingosine-1-phosphate receptor (S1PR) signaling during angiogenesis to enable the dynamic cell-cell junction formation during anastomosis (Hübner et al., 2018). However, at later stages S1PR signaling regulated BBB maturation and VE-cadherin stabilization (Hübner et al., 2018). Interesting, the E3 ubiquitin ligase PDZ domain–containing ring finger 3 (Pdzrn3), which regulates Dvl3 ubiquitinylation, regulated endothelial intercellular junction integrity. Endothelial cell-specific overexpression of Pdzrn3 led to early embryonic lethality with severe hemorrhaging and altered organization of endothelial intercellular junctions (Sewduth et al., 2017). Treatment of the human umbilical vein endothelial cells (HUVEC) with Wnt3a, significantly increased the level of β-catenin and this upregulation was higher when Pdzrn3 was depleted by siRNA and reduced in Pdzrn3 lentiviral-transduced endothelial cells (Sewduth et al., 2014). Very interestingly, treatment of HUVEC with Wnt5a increased the level of p-c-jun which was impaired in Pdzrn3-depleted cells. In addition, Wnt5a treatment induced AP1 response in HUVEC and this induction was repressed by siPdzrn3-treatment and induced in Pdzrn3 lentiviral-transduced endothelial cells (Sewduth et al., 2014). These results outline the regulatory role of Wnt pathway in temporally linking angiogenesis to BBB formation and in anastomosis (Hübner et al., 2018).

### Implication in BBB Formation

The canonical Wnt pathway was shown to act as master regulator of BBB formation and maturation during ontogeny (Liebner et al., 2008). Initial reports showed that β-catenin in human cerebral microvascular endothelial cells (hCMEC/D3) was capable of binding directly to the promoter region of the multidrug resistance protein-1 (MDR1; i.e., ATP-binding cassette sub-family B member-1, ABCB1) gene, a marker of BBB functionality (Pinzón-Daza et al., 2014). In Ctnnb1^iΔEC^ transgenic mice, expression of the tight junction protein claudin-5, glucose transporter-1 (GLUT1), and the major facilitator superfamily domain-containing protein 2 (MFSD2A) a key lipid transporter expressed in brain endothelial cells that is vital for brain lipid uptake, were all significantly decreased, associated to defective BBB structural and functional integrity (Martowicz et al., 2019). The stimulation of hCMEC/D3 endothelial cells with Wnt activators increased the gene expression of the multidrug resistance-associated protein-1 (MRP1; ATP-binding cassette sub-family C member-1, ABCC1), as well as ROCK, whereas Dkk1 induced opposite effects, thus suggesting that the biological activities of the canonical and non-canonical Wnt pathways vary in response to Wnt activators and inhibitors (Pinzón-Daza et al., 2014). Administration of Wnt3aCM in the brain of mice upregulated the expression of the tight junction protein claudin-3, whereas it downregulated the expression of plasmalemma vesicle associated protein (PLVAP), which is involved in endothelial cell fenestration and permeability (Liebner et al., 2008). Furthermore, Wnt7a has been shown to regulate the expression of the BBB-specific GLUT1 in purified endothelial cells *in vitro*, and that β-catenin was required to mediate GLUT1 expression *in vivo* (Engelhardt, 2003). The conditional activation or deactivation of endothelial β-catenin *in vivo* confirmed latter’s requirement in regulating the expression of ABCB1, claudin-3, and PLVAP at the BBB (Liebner et al., 2008). The results showed that the transcriptional activity of β-catenin is necessary and sufficient to upregulate the expression of claudin-3, ABCB1 and downregulate PLVAP in brain endothelial cells, thus inducing the structural and functional properties of the BBB (Liebner et al., 2008). Interestingly, the canonical Wnt pathway is essential for maintaining BBB integrity in adulthood as well. Indeed, using a transgenic mouse model with tamoxifen-inducible endothelial cell-restricted disruption of Ctnnb1 (iCKO), it has been shown that β-catenin depletion in endothelial cells caused neuronal damage and multiple intra-cerebral petechial hemorrhages associated to a downregulation of the tight junction proteins claudin-1 and -3 in the adult brain endothelial cells (Yi et al., 2015).

### Implication in Immunity

Several findings have outlined the role of Wnt pathway in regulating different immune functions. In cultured human aortic endothelial cells (HAEC), exposure to Wnt5a has been shown to increase the level of cyclooxygenase-2 (COX2) (Kim et al., 2010). Wnt3a ligand was reported to exert an anti-inflammatory effects in murine mycobacteria-infected macrophages, and mycobacterium tuberculosis-infected macrophages, notably by regulating the expression of tumor necrosis factor (TNF) (Neumann et al., 2010). Wnt pathway plays an important role in dendritic cells (DCs) differentiation (Zhou et al., 2009). More precisely, β-catenin-TCF/LEF signaling has been demonstrated to interact with Notch signaling to promote the differentiation of DCs (Zhou et al., 2009). Interestingly, activation of the non-canonical Wnt pathway mediated by Wnt5a attenuated DCs differentiation (Zhou et al., 2009). Wnt pathway has been shown to promote as well immune tolerance in DCs (Swafford and Manicassamy, 2016). DCs play an important role in regulating the balance between inflammatory and regulatory responses in the periphery. In LRP5/6^iΔDC^ mice, in which LRP5 and LRP6 are specifically depleted in DCs, the mRNA levels of interleukin (IL)17A, IL22, TNFα increased, whereas the mRNA levels of IL10 and TNFβ were reduced (Suryawanshi et al., 2015). In β-cat^ΔDC^ mice, which specifically lack β-catenin expression in DCs, a higher frequency of T helper 1 (Th1), Th17, TNFα^+^;CD4^+^ T cells, and TNFα^+^;CD8^+^ T cells was observed (Manicassamy et al., 2010). The stimulation of DCs with Wnt3a has been shown to limit the expression of pro-inflammatory cytokines and to increase the expression of anti-inflammatory factors in response to *M. tuberculosis* (Suryawanshi et al., 2015). The Wnt pathway could modulate the immune response through the interaction with other signaling pathways. Indeed, a cross-regulation between the Wnt and nuclear factor-kappa B (NF-κB) signaling cascades has been demonstrated, showing that β-catenin exerted an anti-inflammatory effect by physically inhibiting the NF-κB-mediated transcription of pro-inflammatory genes (Ma and Hottiger, 2016). Wnt5a seems to elicit pro-inflammatory responses via Fzd5, whereas Wnt3a-Fzd1 signaling elicited anti-inflammatory responses (Schaale et al., 2011). Nonetheless, in cultured mouse microglial cells that express the Fzd4/5/7/8 receptors as well as LRP5/6, Wnt3a stimulation activated β-catenin signaling, increasing the expression of pro-inflammatory mediators such as IL6, IL12, and TNFα (Halleskog et al., 2011). However, recent findings have demonstrated that deactivation of the β-catenin signaling induced a pro-inflammatory phenotype in microglial cells (Van Steenwinckel et al., 2019). Interestingly, delivery into the brain of a Wnt agonist that mediates canonical pathway activation specifically in microglia by using a microglia-specific targeting nano-carrier, microglial cell pro-inflammatory phenotype was attenuated (Van Steenwinckel et al., 2019). More investigations are required to clearly address the complex role of Wnt pathway in regulating microglial cell activation.

## The Wnt Pathway in Ischemic Stroke

Stroke constitutes a leading cause of death and long-term disability in adults in the industrialized world. Ischemic stroke accounts for the majority of cases, approximately 85%, and occurs when the cerebral blood flow (CBF) is interrupted due to the sudden obstruction of a cerebral artery caused by an embolus or thrombus (Dirnagl et al., 1999; Moskowitz et al., 2010). The disruption of the regional cerebral blood supply initiates a cascade of events that evolve following three major phases (Dirnagl et al., 1999; Moskowitz et al., 2010). The acute phase, which takes place minutes upon occlusion, is characterized by BBB disruption caused by MMPs activation, oxidative stress, and excitotoxicity, leading to neuronal dysfunction and death (Dirnagl et al., 1999; Moskowitz et al., 2010; ElAli, 2016). The second phase, which takes place hours and days upon occlusion, is characterized by apoptosis, neuroinflammation and exacerbation of BBB breakdown, contributing to the secondary progression of injury (Dirnagl et al., 1999; Moskowitz et al., 2010; ElAli, 2016). The third phase, which takes place days and weeks following upon occlusion, is characterized by the activation of various reparative and regenerative processes that include neuronal plasticity, neurogenesis, angiogenesis and tissue scarring (Dirnagl et al., 1999; Moskowitz et al., 2010; ElAli, 2016). The severity of the early pathological events decelerates brain recovery in the chronic phase, thus significantly worsening outcomes (Dirnagl et al., 1999; Moskowitz et al., 2010). Importantly, ischemic stroke results in two major zones of injury, the infarct core associated to a dramatic reduction of the CBF causing immediate cell death by necrosis, and the peri-infarct penumbra associated to a moderately reduced CBF causing neuronal paralysis that could evolve to cell death (Lo, 2008). Currently, recombinant tissue-plasminogen activator (rtPA)-induced thrombolysis constitutes the only food and drug administration (FDA) approved approach used in clinics to restore CBF (Lo, 2008). The recent evidence is suggesting that Wnt pathway is implicated in ischemic stroke pathobiology, outlining its potential as novel target for the development of new therapeutic interventions.

### Implication in Ischemic Stroke Pathobiology

Several studies have suggested that Wnt pathway is regulated in ischemic stroke patients. The Ischemic Stroke Genetics Study (ISGS) has identified some genetic variants in LRP6 to play a role in determining the risk of ischemic stroke (Harriott et al., 2015). Furthermore, the levels of Dkk1 in the plasma of patients with acute ischemic stroke have been reported to significantly increase in comparison to healthy individuals, as well as patients with stable cerebrovascular disease (Seifert-Held et al., 2011). Interestingly, Dkk1 plasma levels were higher in patients with stable cerebrovascular disease when compared to healthy individuals (Seifert-Held et al., 2011). These findings were confirmed in another independent study showing that the serum levels Scl and Dkk1 were significantly higher in patients with ischemic stroke caused by large artery atherosclerotic (LAA) or small-artery occlusion (SAO) stroke (He et al., 2016) in comparison to healthy individuals. However, no difference in the serum levels of Scl and Dkk1 were detected between the stroke sub-types (He et al., 2016). Both studies did not detect any correlation between Scl or Dkk1 levels and stroke severity or outcome. Nonetheless, recent evidence is suggesting that the elevated levels of serum Dkk1 at baseline were associated with poor prognosis 1 year after ischemic stroke, suggesting that initial Dkk1 levels in the serum could constitute a biomarker for ischemic stroke prognosis (Zhu Z. et al., 2019). In a recent study, miR-150-5p, a regulator of β-catenin and thereby the canonical Wnt pathway, was shown to be upregulated in the blood plasma of hospitalized patient with cerebral infarction (Sun et al., 2020).

The overwhelming experimental findings are indicating that the Wnt pathway is potently and dynamically regulated upon ischemic stroke and is critically involved in disease’s pathogenesis. For instance, Wnt1 levels have been reported to significantly increase 1 to 6 h within the penumbra of rodents subjected to middle artery occlusion (MCAo) (Chong et al., 2010). The re-emergence of the canonical Wnt pathway activity was interpreted as an intrinsic compensatory mechanism to preserve brain homeostasis upon injury (Chong et al., 2010). Indeed, our group has recently demonstrated that β-catenin levels significantly increased in the brain endothelial cells as early as 3 h after MCAo (Jean LeBlanc et al., 2019). Importantly, the early deactivation of the pathway using the potent Wnt inhibitor XAV939 exacerbated BBB breakdown and edema formation, indicating that the pathway re-emerged to preserve BBB structure and function upon injury (Jean LeBlanc et al., 2019). Following the early activation of the pathway, a tendency toward deactivation has been reported and was associated to an upregulation of GSK3β (Jean LeBlanc et al., 2019), and a downregulation of β-catenin and Dvl (Xing et al., 2012) as well as Wnt3a (Wei et al., 2018) within the infarct region. Furthermore, Dkk1 expression was detected within the ischemic region as early as 3 h after MCAo and continued to steadily increase during the following hours (Mastroiacovo et al., 2009). Dkk1-induced expression was correlated with reduced levels of β-catenin in ischemic neurons, outlining Dkk1 potency in modulating canonical Wnt pathway (Mastroiacovo et al., 2009). In the SVZ, β-catenin and Wnt3a expression was shown to decrease during the sub-acute phase after ischemic stroke (Wei et al., 2018). In LRP6^+/–^ mice, which are LRP6 haplo-insufficient, a larger infarct and severe motor deficits as well as increased inflammatory gene expression were reported after MCAo (Abe et al., 2013). Interestingly, Gpr124^flox/–^ mice, which are deficient for the endothelial-specific G-protein-coupled receptor (GPCR) Gpr124, exposed to MCAo exhibited rapid BBB breakdown accompanied by hemorrhagic transformation (Chang et al., 2017). Importantly, BBB breakdown following MCAo was potently rescued in Gpr124^flox/–^Ctnnb1^lox(ex3)/+^;Cdh5-Cre^ERT2^ mice in which β-catenin signaling is specifically activated in endothelial cells (Chang et al., 2017). The induced activation of β-catenin signaling in Gpr124^flox/–^ mice attenuated hemorrhagic transformation and restored the pericyte-endothelial cell crosstalk (Chang et al., 2017). Interestingly, Wnt7a/b, two of the most potent factors implicated in physiological angiogenesis are not activated during post-stroke angiogenesis, suggesting that the latter does not constitute a simple recapitulation of developmental angiogenesis (Buga et al., 2014). Transcriptomic studies have shown that the proliferation of vascular smooth muscle cells (VSMCs), which contribute to post-stroke angiogenesis, is modulated by LEF1 and Wnt4a, and that this modulation was more important in young animals compared to aged animals after MCAo, whereas Wnt5 was specifically increased in the brain of aged animals (Buga et al., 2014). Furthermore, the deposition of the extracellular matrix proteins (ECM) limits the plasticity and remodeling capability of the microvasculature within the scarring zone upon MCAo, was exacerbated in aged animals (Johnson and DiPietro, 2013), accompanied by an increased expression of Wnt5b gene in the peri-lesional region in the cortex (Buga et al., 2014). Dkk1 is induced in neurons within the infarct core and the penumbra and was associated to a reduced expression of β-catenin (Mastroiacovo et al., 2009). Treatment with lithium ions rescued canonical Wnt pathway activity and was highly protective against ischemia (Mastroiacovo et al., 2009). Importantly, doubleridge mice, which have a reduced basal expression of Dkk1, showed an attenuated reduction of β-catenin and a reduced infarct volume following MCAo, providing a direct proof that Dkk1 contributes to the injury progression in ischemic stroke (Mastroiacovo et al., 2009). It is well established that 17β-estradiol (i.e., E2, estrogen) exerts neuroprotective effects following cerebral ischemia (Zhang et al., 2008). E2 mediates its neuroprotective effects, at least partly, by attenuating the post-ischemic induction of Dkk1 (Zhang et al., 2008). Indeed, E2 reduced ischemia-induced Dkk1 expression, which correlated with elevated levels of nuclear β-catenin, and enhanced the expression of Wnt3 at the lesion site (Zhang et al., 2008). These effects were associated as well to the modulation of JNK/c-Jun signaling (Zhang et al., 2008). It is well known that the pre-menopausal women are somehow protected against ischemic stroke in comparison to age-matching men (Roquer et al., 2003). This tendency is drastically inverted after menopause, and aged women have worse stroke outcomes when compared with men (Di Carlo et al., 2003). The functional link between E2 and Wnt pathway via Dkk1 is highly interesting, as it might accounts for the sex-dependent disparities observed in ischemic stroke recovery, thereby constituting a novel target for the development of therapeutic interventions that are tailored for the biological sex.

**FIGURE 1:**
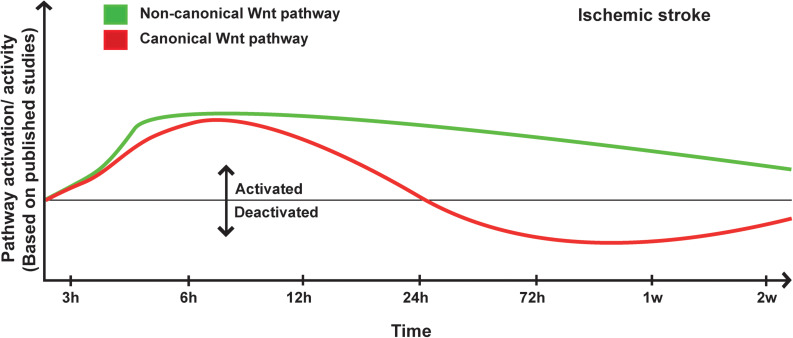
Proposed scheme for Wnt pathway regulation in ischemic stroke. A representation of canonical and non-canonical Wnt pathways temporal regulation following ischemic stroke onset based on pathway activation (i.e., modulation of pathway’s key components) and activity (i.e., regulation of specific target genes) as reported in the different published studies.

The accumulating evidence is indicating that the non-canonical Wnt pathway is deregulated in ischemic stroke (Wang and Liao, 2012). Among the various signaling components, ROCK pathway seems to play a particularly important role. Indeed, in global hemizygous ROCK2^+/–^ and endothelial-specific (EC-ROCK2)^–/–^ mice, endothelial nitric oxide synthase (eNOS) mRNA stability and expression were increased after MCAo (Hiroi et al., 2018). This was correlated with an enhanced endothelium-dependent relaxation and neuroprotection (Hiroi et al., 2018). The neuroprotective effects observed in ROCK2^+/–^ were totally abolished upon eNOS depletion (Hiroi et al., 2018). These findings indicate that ROCK2 plays important role in regulating eNOS expression and NO-mediated maintenance of the neurovascular functions after ischemic stroke (Hiroi et al., 2018). In a recent study, the depletion of profilin-1 (Pfn1), which is an actin-binding protein involved in the dynamic transformation and reorganization of cytoskeleton, attenuated damage upon cerebral ischemia via modulation of microglial cell function associated with the RHOA/ROCK pathway (Lu et al., 2020). Furthermore, it has been shown that JNK and c-Jun phosphorylation is increased very early within the infarct core and peri-lesional region in the brain of rats after MCAo (Ferrer et al., 2003). Moreover, in JNK1^–/–^ mice the infarct size is exacerbated following MCAo (Brecht et al., 2005). The expression of TNF receptor-associated factor-6 (TRAF6) was markedly increased after cerebral ischemia in mice (Li T. et al., 2017). TRAF6 induced RAC1 activation and consequently exacerbated ischemic injury by directly binding and ubiquitinating RAC1 (Li T. et al., 2017). TRAF6 depletion reduced the infarct volume and ameliorated the neurological functions following MCAo (Li T. et al., 2017). TRAF6 depletion attenuated the pro-inflammatory response, oxidative stress, and neuronal death (Li T. et al., 2017). More investigations are still needed to decipher and fully address the complex role of the non-canonical Wnt pathway in ischemic stroke pathobiology (Figure 1).

### Implication in Ischemic Stroke Therapy

In anticipation of its potential as a major therapeutic target, there is currently a growing interest in investigating the impact of Wnt pathway modulation on structural and functional recovery after ischemic stroke. Ischemic stroke induces neurogenesis in the SVZ where stem/progenitor cells shift from asymmetric to symmetric cell division, and migrate toward the peri-lesional zone in attempt to integrate and replace the lost neurons (Marchetti and Pluchino, 2013). Moreover, NPCs have been shown to use the vasculature as scaffold to migrate to the lesion site within specialized neurovascular niches (Ohab et al., 2006; Hermann and Chopp, 2012; ElAli et al., 2014). Ischemic stroke induces as well angiogenesis within the lesion site as an attempt to ameliorate cerebral blood perfusion, enhance the uptake of nutrients, and promote the secretion of neurotrophic factors (Ohab et al., 2006; Hermann and Chopp, 2012). Unfortunately, these responses do not improve recovery after stroke, as NPCs have slow proliferation rate, newborn neurons do not survive the ischemic “milieu,” and density of the new microvasculature is not sufficient to adequately perfuse the injured tissue (Ohab et al., 2006; Wu et al., 2017). These processes are associated to an activation of the canonical Wnt pathway, translating an intrinsic attempt from the brain to repair itself via re-emergence of specialized developmental mechanisms (Piccin and Morshead, 2011; Hermann and ElAli, 2012). It has been proposed that amplification of the Wnt pathway activity would allow boosting the endogenous protective and restorative processes. Indeed, the exogenous delivery of recombinant Wnt3a into the brain of mice improved short- and long-term tissue repair and regeneration following MCAo (Wei et al., 2018). This was attributed to an upregulation of the brain-derived growth factor (BDNF), and stimulation of the proliferation and migration of NPCs from the SVZ toward the peri-lesional zone, thus increasing the number of newborn neurons at the injured site (Wei et al., 2018). Additionally, Wnt3a administration enhanced the regional CBF within the peri-lesional zone (Wei et al., 2018). Importantly, Dkk1 abolished most of the beneficial effects of Wnt3a administration (Wei et al., 2018). MiRNA-148b has been reported to be overexpressed in the SVZ of rats upon MCAo (Wang et al., 2017). MiRNA-148b has been shown to suppress the expression of Wnt1 and β-catenin, hence acting as negative regulator of the canonical Wnt pathway (Wang et al., 2017). Inhibition of miRNA-148b expression using a LV-miR-148b inhibitor promoted the proliferation and differentiation of NSCs into neurons and astrocytes, and improved functional recovery (Wang et al., 2017). These effects were abolished upon depletion of Wnt1 (Wang et al., 2017). The delivery of LV-Wnt3a-HA directly into the striatum of mice enhanced long-term functional recovery after cerebral ischemia, and increased the number of bromodeoxyuridine (BrdU)^+^ cells that differentiated into mature neurons in the ischemic striatum (Shruster et al., 2012). On the other hand, delivery of LV-Wnt3a-HA into the SVZ enhanced functional recovery early after cerebral ischemia and increased the number of immature neurons in the striatum as well as SVZ, accompanied by attenuation of neuronal injury (Shruster et al., 2012). The delayed effects following intra-striatal LV-Wnt3a-HA administration, up to 1 month after ischemia, suggest that the observed functional recovery was not attributable to the rescuing of pre-existing damaged neurons but rather to the improved neurogenesis within the neurogenic niches (Shruster et al., 2012). This might be attributed to Dkk1-induced expression within the ischemic striatum, which prevented Wnt3a from binding to LRP5/6 (Shruster et al., 2012). Galangin, a natural flavonoid isolated from the rhizome of *Alpinia officinarum Hance*, has been shown as well to improve neurovascular functions after cerebral ischemia, and to ameliorate neurological functions through activation of the canonical Wnt pathway coupled with hypoxia-inducible factor-1α (HIF1α) and vascular endothelial growth factor (VEGF) (Wu et al., 2015). Interestingly, hypoxic post-conditioning (HPC) increased the levels of nuclear β-catenin and the expression of Wnt3a, while decreasing the expression of Dkk1 after cerebral ischemia in rats (Zhan et al., 2019). The effects of HPC were abolished by the LV-mediated overexpression of Dkk1 in the brain (Zhan et al., 2019). HPC reduced the activity of GSK3β, an effect that was recapitulated following the pharmacological inhibition of GSK3β using SB216763 (Zhan et al., 2019). These results suggest that activation of the canonical Wnt pathway through Dkk1 inhibition and GSK3β deactivation jointly contribute to the neuroprotective effects of HPC against ischemic injury (Zhan et al., 2019). Moreover, the acute administration of 4,6-disubstituted pyrrolopyrimidine (TWS119), a specific inhibitor of GSK3β, has been shown to provide neuroprotection by decreasing the infarct volume and reducing BBB permeability after cerebral ischemia in rodents (Wang W. et al., 2016). These effects were associated to canonical Wnt signaling pathway activation, which increased the expression of tight junction proteins claudin-3 and ZO1 (Wang W. et al., 2016). In parallel, the delayed administration of TWS119 has been shown to improve long-term neurological functions associated to enhanced angiogenesis and neural plasticity after ischemic stroke (Song et al., 2019). Furthermore, pathway delayed activation enhanced the expression of PSD95, the pre-synaptic marker synaptophysin, as well as the growth-associated protein-43 (GAP43) (Song et al., 2019). Additionally, canonical Wnt pathway delayed activation stimulated microglia cell polarization toward a reparative phenotype in the sub-acute phase translated by an increased expression of CD206, Arginase-1 (Arg1) and chitinase3-like 3 (YM1/2) (Song et al., 2019). This was accompanied by an enhanced expression of various anti-inflammatory mediators, including IL10 and TGFβ (Song et al., 2019). Indeed, previous studies have shown that Wnt3a induced an anti-inflammatory M2-like phenotype in microglial cells via canonical Wnt pathway activation, which increased the expression of Arg1 (Matias et al., 2019). In this regard, the delivery of Wnt3a attenuated the inflammatory response upon MCAo by modulating microglial cell activation and phenotype (Zhang et al., 2019). Wnt3a administration downregulated the expression of pro-inflammatory markers, such as the inducible NOS (iNOS) and TNFα, whereas upregulated the expression of anti-inflammatory markers, such as CD206 and Arg1 (Zhang et al., 2019). The immunomodulatory potential of canonical Wnt pathway activation could be associated to an activation of autophagy through Beclin-1 and the microtubule-associated protein light chain-3 (LC3)-II (Zhou et al., 2011). Ischemic stroke strongly induces the activation of astrocytes that contribute to scar formation, thus physically separating the injured tissue from the intact region (Liu and Chopp, 2016). Importantly, Wnt3a treatment was efficient in decreasing the number of neurotoxic activated astrocytes (A1 phenotype) and to increase the number of neuroprotective activated astrocytes (A2 phenotype), via reduction of glial fibrillary acidic protein (GFAP) expression (Zhang et al., 2019). This was accompanied by reducing the expression of IL15, which induces neurotoxic glial activation, and by increasing the expression of IL33, which promotes neuroprotective glial activation (Zhang et al., 2019). In a previous study, it has been reported that administration of Sulindac, a non-steroidal anti-inflammatory drug, in rats after MCAo activated the canonical Wnt pathway by inducing the expression of Dvl and β-catenin, and provided anti-apoptotic effect by increasing the expression of B-cell lymphoma-2 (BCL2) and decreasing the expression of Bcl-2-associated X (BAX) in the ischemic brain (Xing et al., 2012). Importantly, BCL2 has been shown to stimulate neurogenesis in rats upon MCAo by inhibiting the function of bone morphogenetic protein-4 (BMP4), which has been shown to negatively regulate adult brain neurogenesis and to direct neural progenitors to a glial fate, via activation of β-catenin signaling (Lei et al., 2012). In a recent study, it has been shown that the transplantation of oligodendrocyte precursor cells (OPCs) in the brain of mice after MCAo reduced the infarct and edema volumes, and improved the neurological functions (Wang et al., 2020). OPCs transplantation attenuated BBB breakdown by increasing the expression of claudin-5 and occludin (Wang et al., 2020). These effects were mediated via the activation of the canonical Wnt pathway, as OPCs transplantation increased the expression of β-catenin and Wnt7a in the ischemic brain (Wang et al., 2020). Pharmacological inhibition of the canonical Wnt pathway totally abolished the beneficial effects of OPCs transplantation (Wang et al., 2020).

As mentioned earlier, ischemic stroke induces BBB breakdown, which promotes complications such as edema formation and inflammation (ElAli et al., 2011; Jean LeBlanc et al., 2019). Currently, thrombolysis via rtPA administration constitutes the only existing approach approved by the FDA to treat acute ischemic stroke (Jean LeBlanc et al., 2019). rtPA significantly enhances stroke outcomes by restoring CBF to the ischemic region (Wang et al., 2004; Jean LeBlanc et al., 2019). However, rtPA should be administered within a narrow therapeutic window of 4.5 h after onset due to the elevated risk of causing hemorrhagic transformation (HT), a life-threatening post-stoke complication (Sussman and Connolly, 2013). Therefore, only around 5% of eligible stroke patients could benefit from thrombolysis (Wang et al., 2004). It has been demonstrated that HT associated to rtPA administration is mediated via the activity of MMPs, namely MMP9, which exacerbates BBB breakdown via excessive degradation of the ECM and tight junction proteins (Wang et al., 2004). Furthermore, our group has recently demonstrated that rtPA increased the hypoxia-induced expression of PLVAP, increasing vascular permeability and leakage (Jean LeBlanc et al., 2019). Interestingly, the administration of the GSK3β inhibitor, TWS119, to activate the canonical Wnt pathway attenuated rtPA-induced HT in rats upon MCAo. Indeed, TWS119 administration reduced BBB breakdown and improved structural and functional recovery by increasing the expression of claudin-3, and ZO1 (Wang W. et al., 2016). These results suggest that pathway activation could prevent tPA-induced HT after acute ischemic stroke. However, in this study the pathway was activated 4 h after MCAo, thus within the approved therapeutic window for rtPA administration, limiting its clinical relevance. Our group has recently demonstrated that canonical Wnt pathway activation using a potent agonist, 6-bromoindirubin-3′-oxime (6-BIO), attenuated BBB breakdown and reduced the incidence of HT associated to delayed rtPA administration (Jean LeBlanc et al., 2019). In this study, rtPA was administered 6 h after MCAo, thus beyond the current therapeutic window. Canonical pathway activation, which was translated by increased levels of β-catenin restored the expression of claudin-3 and claudin-5, and attenuated the basal endothelial permeability by repressing PLVAP expression (Jean LeBlanc et al., 2019). These findings suggest that activation of the canonical Wnt pathway could extend the therapeutic window of rtPA via attenuation of BBB breakdown (Jean LeBlanc et al., 2019).

Using a potent cell-penetrating peptide D-JNKI1, which selectively block the access of JNK to c-Jun, it has been shown that deactivation of JNK/c-Jun pathway significantly attenuated NMDA receptor-mediated excitotoxicity and subsequent cell death in the brain of rodents after MCAo (Borsello et al., 2003). In another study, D-JNKI1 has been demonstrated to markedly prevent c-Jun phosphorylation after MCAo in rats, in the core and the peri-lesional zone, resulting in a strong inhibition of caspase-3 activation in the core (Repici et al., 2007). The administration of JNK-IN-8, a potent JNK inhibitor with high specificity, improved structural and functional recovery through suppressing of neuroinflammation in the brain of rats following MCAo (Zheng et al., 2020). JNK-IN-8 administration exerted anti-inflammatory effects by attenuating the activation of microglia and reducing expression of IL6, IL1β, and TNFα expression (Zheng et al., 2020). Furthermore, JNK-IN-8 suppressed the activation of NF-κB signaling, translated by reduced levels of p65 (Zheng et al., 2020). These findings suggest that deactivation of the non-canonical Wnt pathway provided protection in the context of ischemic stroke. Indeed, it is well established that ROCK, which is a main downstream effector in the non-canonical Wnt pathway, plays a negative role in ischemic stroke (Sladojevic et al., 2017). The administration of fasudil and Y-27632, which are potent ROCK inhibitors, has been shown to ameliorate the CBF to both ischemic and non-ischemic brain of mice after MCAo, reduced infarct size and improved neurological functions (Rikitake et al., 2005). These observations were associated to a reduced ROCK activity within the vasculature as well as brain parenchyma, and an increased eNOS expression and activity (Rikitake et al., 2005). The protective effects of ROCK inhibition were abolished upon eNOS genetic depletion in mice (Rikitake et al., 2005). ROCK inhibition using fasudil after MCAo in rats reduced infarct size, neuronal apoptosis as well as caspase-3 activity, subsequently improving neurological recovery (Wu J. et al., 2012). ROCK has been shown to mediate inflammation, thrombosis formation, and vasospasm by affecting the function of vascular and inflammatory cells (Wang and Liao, 2012). For instance, peripheral leukocyte ROCK activity was reported to increase in acute stroke patients compared to healthy individuals with maximal activity occurring about 48 h after stroke onset (Feske et al., 2009). ROCK inhibition using Y-27632 after MCAo in rats decreased infarct size, reduced oxidative stress and alleviated the inflammatory response in the ischemic brain (Li and Liu, 2019). ROCK inhibition attenuated neuronal apoptosis by modulating the expression of caspase-3/8/9 as well as BAX/BCL2 ratio (Li and Liu, 2019). Furthermore, ROCK inhibition attenuated as well the activation of astrocytes and microglial cells at the lesion site (Li and Liu, 2019). Importantly, inhibition of ROCK activity was proposed to account for the pleiotropic non-cholesterol protective effects of statins in ischemic stroke (Sacco and Liao, 2005). For example, some of the beneficial effects of statins in ischemic stroke are attributed to the inhibition of geranylgeranyl pyrophosphate formation required for the function of RHO-GTPases, thus altering RHO/ROCK pathway (Wang and Liao, 2012). Outlining the clinical relevance of ROCK inhibition in ischemic stroke therapies, fasudil was assessed in clinical studies and has been shown to improve the outcomes of ischemic stroke patients when administered within 48 h after onset (Shibuya et al., 2005). Sanggenon C (SC), a natural flavonoid extracted from the Cortex Mori *Sang Bai Pi*, was reported to possess anti-inflammatory and antioxidant properties under hypoxic conditions (Zhao and Xu, 2020). SC administration in rats after MCAo improved structural and functional recovery by reducing inflammation, oxidative stress, and apoptosis (Zhao and Xu, 2020). RHOA overexpression abolished SC properties, indicating that SC mediated its protective effects by inhibiting RHOA/ROCK pathway (Zhao and Xu, 2020).

The recent findings are suggesting that CaMKII activity, which is another major component of the non-canonical Wnt pathway, constitutes a potential target for neuroprotection after ischemic stroke (Coultrap et al., 2011). CaMKII, which has been shown to mediate major effects of physiological NMDA-receptor stimulation, is implicated in the pathological glutamate signaling after ischemic stroke (Vest et al., 2010). Indeed, inhibition of stimulated and autonomous CaMKII activity in the brain of mice after MCAo using tatCN21 attenuated glutamate-mediated neuronal cell death in the ischemic brain (Vest et al., 2010). However, another study has demonstrated that the neuroprotection observed upon CaMKII inhibition could be seen only acutely immediately, whereas a sustained CaMKII inhibition associated to excitotoxicity could exacerbate neuronal death by increasing neuronal vulnerability to glutamate (Ashpole and Hudmon, 2011). Collectively, these findings highlight the promises of targeting the non-canonical Wnt pathway in ischemic stroke (Supplementary Table 1).

## The Wnt Pathway in Hemorrhagic Stroke

Hemorrhagic stroke, which comprises essentially intra-cerebral hemorrhage (ICH) and subarachnoid hemorrhage (SAH), accounts for approximately 15% of stroke cases (Qureshi et al., 2009). Hemorrhagic stroke is a devastating pathological condition as it is more likely to result in fatality or severe disability in survivors (Morioka et al., 2017). During hemorrhagic stroke, a rapid accumulation of blood within the brain parenchyma leads to disruption of the normal anatomy, and increases local pressure (Aronowski and Zhao, 2011). When hemorrhagic volume exceeds 150 mL acutely, cerebral perfusion pressure falls to zero and the patient dies. If the hemorrhagic volume is smaller than 140 mL, most patients survive the initial insult (Xi et al., 2006). ICH evolves within three distinct phases; (1) initial hemorrhage, which is caused by the rupture of cerebral arteries, (2) hematoma expansion, which occurs during the first hours after initial hemorrhage onset and is implicated in the increased intra-cranial pressure that disrupts local tissue integrity and the BBB, and (3) the peri-hematomal edema, which is formed around the hematoma causing secondary insult (Magistris et al., 2013). During the secondary insult, some biological modification appears such as cytotoxicity of blood, hyper-metabolism, excitotoxicity, spreading depression, oxidative stress, inflammation, and exacerbated BBB disruption (Aronowski and Zhao, 2011). This peri-hematomal edema is the primary etiology for neurological deterioration and develops over days following the initial insult that itself can lead to secondary brain injury resulting in severe neurological deficits and sometimes delayed fatality (Xi et al., 2006). Ultimately, this pathogenesis leads to irreversible disruption of the components of the neurovascular unit, leading to deadly brain edema with massive brain cell death. Whereas inflammatory mediators generated locally in response to brain tissue injury have the capacity to increase damage caused by ICH, inflammatory cells are vital for the removal of cell debris from hematoma (Aronowski and Zhao, 2011). In more than 40% of intra-cerebral hemorrhage cases, hemorrhage extends into the cerebral ventricles causing intra-ventricular hemorrhage. This is associated with acute obstructive hydrocephalus and substantially worsening the prognosis (Magistris et al., 2013). The recent evidence is suggesting that Wnt pathway is implicated in hemorrhagic stroke pathobiology, thus outlining its potential as novel target for the development of new therapeutic interventions.

### Implication in Hemorrhagic Stroke Pathobiology

Several studies have suggested that Wnt pathway is deregulated in hemorrhagic stroke. Indeed, in hemorrhagic stroke patients with spontaneous non-traumatic ICH, a decreased level of nuclear β-catenin in brain endothelial cells located near the bleeding site was reported, suggesting a deactivation of the canonical Wnt pathway at the BBB (Tran et al., 2016). Moreover, the protein expression of APC was decreased in the brain of intra-cranial aneurysm type of patients, independently of biological sex and age, and was associated to the intra-cranial aneurysm diameter (Lai et al., 2019). In addition, RHOA expression was significantly increased in the peripheral blood mononuclear cells (PBMCs) on days 0, 2, and 4 after aneurysmal SAH patients (González-Montelongo et al., 2018). Interestingly, a strong correlation between RHOA expression/activity and injury severity was observed in patients at days 2 and 4 (González-Montelongo et al., 2018). There was no significant increase in activated RHOA in patients who developed vasospasm versus patients without vasospasm on day 0 as well as on day 2 whereas active RHOA was significantly increased on day 4 (González-Montelongo et al., 2018).

The overwhelming experimental findings are indicating that Wnt pathway is potently regulated upon hemorrhagic stroke, and is critically involved in disease pathogenesis. Indeed, expression of Norrin, a key protein implicated in BBB formation, which activates Fzd4 receptor, has been shown to significantly increase 6 to 24 h after SAH, which was induced by endovascular perforation in rats (Chen et al., 2015). Aldolase C, a positive regulator of Wnt signaling that acts through destabilization of Axin, significantly increased during early brain injury associated to SAH, subsequently increasing the expression of Axin (Ruan et al., 2020). Following SAH, Wnt1 and Wnt3a levels significantly decreased 12 h after onset (Wang Y. et al., 2019; Ruan et al., 2020), whereas the levels of β-catenin, Fzd1 decreased as early as 6 h after onset (Wang Y. et al., 2019; Ruan et al., 2020). Twenty-four hours after ICH and SAH, the ratio of p-tyrosine-GSK3β/GSK3β (i.e., indicating an enhanced kinase activity) (Krafft et al., 2012, 2013; Zuo et al., 2017), and p-serine-β-catenin/β-catenin were increased (Krafft et al., 2013; Zuo et al., 2017; Li et al., 2018), whereas the ratio p-serine-GSK3β/GSK3β (i.e., indicating a reduced kinase activity) was decreased (Li et al., 2018). Interestingly, another study has reported that in the peri-hematomal β-catenin expression in endothelial cells is downregulated, whereas GSK3β expression and activity remained unchanged (Zhao et al., 2017). On the other hand, Dkk1 expression in the basal ganglia and peri-hematomal remarkably increased during early brain injury associated to ICH, whereas it remains unchanged in the contralateral basal ganglia and in the blood serum (Li Z. et al., 2017; Wang G. et al., 2019). Interestingly, Dkk1 expression was found in the neuron and microglia but not in astrocytes (Wang G. et al., 2019), and correlated with a decreased level of Wnt1 in neurons (Wang Y. et al., 2019). GSK3β was activated as well in early brain injury as well as in the second phase of hemorrhagic stroke within the CA1, CA3, and DG regions of the hippocampus (Liu et al., 2018). Early deactivation of the canonical Wnt pathway using Wnt1 or Fzd1 siRNA worsened brain edema after SAH and exacerbated neurological deficits (Ruan et al., 2020), outlining the importance of canonical Wnt pathway after hemorrhagic stroke. In tamoxifen-inducible endothelial cell-restricted disruption of ctnnb1 (iCKO) mice in which β-catenin is completely depleted, the expression of both claudin-1 and claudin-3 were diminished in brain endothelial cells (Tran et al., 2016). Loss of 60% of claudin-1 expression was associated to an increased permeability of the BBB in iCKO mice, leading to petechial hemorrhage in the brain of mutant mice (Tran et al., 2016).

**FIGURE 2:**
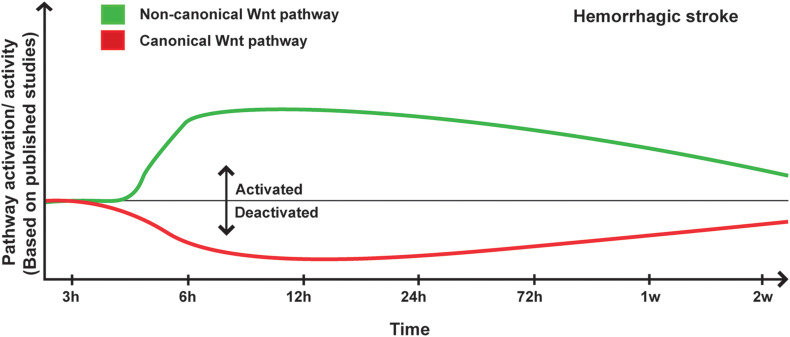
Proposed scheme for Wnt pathway regulation in hemorrhagic stroke. A representation of canonical and non-canonical Wnt pathways temporal regulation following hemorrhagic stroke onset based on pathway activation (i.e., modulation of pathway’s key components) and activity (i.e., regulation of specific target genes) as reported in the different published studies.

Recent studies have demonstrated an important role as well of the non-canonical Wnt in hemorrhagic stroke. For instance, the expression and activity of ROCK and RHOA significantly increased 24 h after SAH (Fujii et al., 2012; Huang et al., 2012; Zhao H. et al., 2016), whereas RAC1, which counteracts the biological activity of RHOA, remained unchanged, correlating with low levels of β-catenin within the adherent junctions (Huang et al., 2012). Ephrin receptor-A4 (EphA4) activation has been shown to induce the phosphorylation of Ephexin1, which preferentially activates RHOA (Fan et al., 2017). Furthermore, EphA4 has been shown to promote cell death and apoptosis (Conover et al., 2000; Lemmens et al., 2013). EphA4 expression increased as well as Ephexin1, RHOA and ROCK2 (Fan et al., 2017) in the brain of rats after SAH, and EphA4 was strongly expressed in neurons, astrocytes, and microglia (Fan et al., 2017). Importantly, hemoglobin (Hb) extravasation after ICH has been demonstrated to exacerbate BBB disruption as well as edema formation within the peri-hematomal region as early as 24 h after onset (Koeppen et al., 1995). Six and 24 h following Hb intra-cerebral injection, RHOA and ROCK2 activities were significantly increased, and ROCK2 activity positively correlated with MMP9 expression levels (Fu et al., 2014). In addition, the purinergic receptor P2X7, which is implicated in modulating BBB integrity, was upregulated following hemorrhagic stroke via the activity of RHOA (Zhao H. et al., 2016). RHOA/ROCK pathway plays key role in mediating the contraction of the VSMCs of the basilar arteries in SAH animals during the early brain injury phase (Egea-Guerrero et al., 2015), as well as in the second phase of hemorrhagic stroke (Naraoka et al., 2013). Moreover, it has been shown that the Wnt/PCP pathway is regulated as well following hemorrhagic stroke, translated by increased levels of JNK and c-Jun phosphorylation at the early stages after onset within the basal ganglia and cortical basal brain (Wan et al., 2009; Ling et al., 2019; Xu et al., 2020, p. 3), as well as within the basilar arteries at least until day 7 after onset (Yatsushige et al., 2005, 2008). However, another study reported no changes in JNK and c-Jun phosphorylation, as well as no correlation with P2X7 upregulation (Wen et al., 2017). The expression of Dkk3, which acts as a negative regulator for the canonical Wnt signaling similarly to Dkk1, and Dvl were downregulated very early after ICH, whereas JNK/AP1 signaling was induced (Xu et al., 2020, p. 3). Interestingly, CaMKII, which is another component of the non-canonical Wnt pathway, plays an important role in the regulation of intracellular Ca^2+^ homeostasis VSMCs contraction and vascular inflammation (House et al., 2008). It has been shown that CaMKII phosphorylation increased 1 h after SAH in the cerebral arteries whereas CaMKII protein expression increased after 3 days within the same arteries (Edvinsson et al., 2014). CaMKIIα, which is one of the major isoforms of CaMKII, was shown to modulate the inflammatory response of microglial cells (Huang et al., 2015). Tyrosine phosphorylation of CaMKIIα, which modulate protein kinase activity, was shown to increase immediately after SAH induction essentially in neurons and microglia within the lesion site (Makino et al., 2015; Zhou et al., 2018) (Figure 2).

### Implication in Hemorrhagic Stroke Therapy

In the recent years, several studies have evaluated the impact of the Wnt pathway modulation on structural and functional recovery after hemorrhagic stroke. It has been proposed that amplifying the activity of the canonical Wnt pathway or attenuating that of the non-canonical Wnt pathway could promote the reparative and restorative processes. Indeed, the exogenous intranasal delivery of Wnt3a into the brain of SAH rats improved short-, mid- and long-term neuronal function and reduced brain edema without influencing SAH severity (Ruan et al., 2020). Administration of the Wnt inhibitor XAV939 counteracted most of the beneficial effect of Wnt3a after SAH (Ruan et al., 2020). On the other hand, the exogenous delivery of recombinant human Wnt1 (rhWnt1) into the ventricle of SAH rats decreased brain edema and ameliorated neurological functions (Wang Y. et al., 2019). Interestingly, these beneficial effects were totally abolished after administration of Wnt1 siRNA or a neutralizing monoclonal antibody anti-Fzd1 (Wang Y. et al., 2019). These results highlight the importance of the canonical Wnt pathway as a therapeutic target to promote neurovascular repair following hemorrhagic stroke. In both studies, SAH was associated to a deactivation of the canonical Wnt pathway translated by reduced levels of Wnt1, Wnt3a and Fzd1. In this regard, the intraperitoneal delivery of 6-BIO, a GSK3β inhibitor, improved neurological functions, reduced brain hematoma volume, and stimulated regeneration after ICH (Zhao et al., 2017). This was attributed to an upregulation of BDNF, which stimulates the proliferation and migration of neuronal progenitors from the SVZ toward the injured zone, thus increasing the number of newborn neurons within in the peri-hematomal brain region (Zhao et al., 2017). 6-BIO also improved angiogenesis by an increase number of BrdU^+^/GLUT1^+^ cells in the peri-hematomal zone during the second phase of hemorrhagic stroke (Zhao et al., 2017). As mentioned, the expression Dkk1, a potent endogenous inhibitor of the canonical Wnt pathway, increased in the basal ganglia early after ICH (Li Z. et al., 2017), and was expressed essentially in neurons, and microglia (Wang G. et al., 2019). The administration of Dkk1 siRNA into the brain ventricles of rats attenuated BBB disruption after ICH by increasing the expression of ZO1, consequently decreasing brain edema and attenuating neurological deficits (Li Z. et al., 2017). These results suggest that Dkk1 neutralization is neuroprotective against the secondary injury following ICH, and that the underlying mechanisms are associated with an improved integrity of the BBB (Li Z. et al., 2017). These observations might be associated with enhanced biological activities of Wnt1 or Wnt3a, as Dkk1 prevents Wnt1 and Wnt3a binding to LRP5/6 (Shruster et al., 2012). In this regard, our group has recently demonstrated that deactivation of the canonical Wnt pathway using XAV939 increased the risk of spontaneous intra-cerebral HT following ischemic stroke by exacerbating BBB permeability (Jean LeBlanc et al., 2019). Furthermore, minocycline, a semi-synthetic tetracycline derivative, alleviated the severity of brain edema and BBB disruption, thus ameliorating neurological functions, notably by upregulating the expression of occludin in a rodent collagenase-induced ICH model (Wang G. et al., 2019). Minocycline activated the canonical Wnt pathway by increasing the abundance of β-catenin and Wnt1, and reducing the expression of Dkk1, thereby inducing the expression of the tight junction occludin (Wang G. et al., 2019). Interestingly, the administration Dkk1 siRNA amplified the protective effects of minocycline (Wang G. et al., 2019). These results suggest that reducing Dkk1 expression constitutes an interesting strategy to attenuate brain damage after hemorrhagic stroke (Wang G. et al., 2019). Norrin is a secreted protein that plays an important role in regulating angiogenesis via activation of the Fzd4, a receptor implicated in canonical Wnt pathway (Chen et al., 2015). Exogenous delivery of recombinant Norrin (rNorrin) into the ventricles of rats after SAH has been shown to provide neuroprotection by reducing brain edema and attenuating BBB permeability via activation of the canonical Wnt pathway, translated by an increased level of nuclear β-catenin (Chen et al., 2015). This was associated to an increased expression of tight junction proteins, namely occludin, ZO1, and VE-cadherin (Chen et al., 2015). Interestingly, Fzd4 siRNA prophylactic treatment attenuated the beneficial effects of rNorrin (Chen et al., 2015). The α7 nicotinic acetylcholine receptor (α7nAChR) has been shown to activate the phosphatidylinositol 3-kinase (PI3K)/Akt signaling pathway, which mediates GSK3β inhibition and thus β-catenin stabilization (Moccia et al., 2004). Importantly, functional α7nAChR was detected in the cerebral microvasculature (Moccia et al., 2004), and its activation attenuated BBB disruption after hemorrhagic stroke (Krafft et al., 2013). Indeed, PHA-543613, an agonist of α7nAChR, decreased GSK3β expression and subsequently stabilized β-catenin, reducing the peri-hematomal brain edema and improving the BBB functional integrity by increasing the expression of claudin-3 and claudin-5 (Krafft et al., 2013). On the other hand, administration of the GSK3β inhibitors, lithium and TWS119, attenuated the sensorimotor deficits and reduced brain edema by increasing β-catenin nuclear expression, which upregulated claudin-1 and claudin-3 expression, consequently improving BBB integrity after ICH (Li et al., 2018). Moreover, lithium reduced the number of OX6-positive cells microglia in the peri-hematomal zone as well as decreased the expression of the pro-inflammatory mediator COX2 during the early brain injury phase (Kang et al., 2012). M1-type microglia promote the inflammatory response by releasing pro-inflammatory mediators, such as TNFα, IL1β, and IL6, exacerbating tissue damage (Ueba et al., 2018), whereas M2-type microglia exert protective effects by promoting the release of anti-inflammatory mediators and trophic factors, and contribute to tissue repair (Yang et al., 2018). Increasing the brain levels of Wnt1 via the delivery of rhWnt1 alleviated early brain injury associated to SAH in rats, which was accompanied by increased expression of β-catenin (Wang Y. et al., 2019). Activation of the canonical Wnt pathway via rhWnt1 delivery stimulated microglia cell polarization toward a M2-type reparative phenotype during the early brain injury phase by increasing the expression of CD36, CD206 and peroxisome proliferator-activated receptor-γ (PPARγ), and decreasing the protein levels of NF-κB (Wang Y. et al., 2019). Delivery of rhWnt1 reduced as well the release of pro-inflammatory cytokines such as IL1β, IL6, and TNFα (Wang Y. et al., 2019). In contrast, administration Wnt1 siRNA or neutralizing monoclonal antibody anti-Fzd1 resulted in opposite effects (Wang Y. et al., 2019). The intranasal injection of Wnt3a in rats after SAH activated the canonical Wnt pathway translated by the increased expression of β-catenin, Fzd1, aldolase C, PPAN, and the decreased expression of Axin (Ruan et al., 2020). Wnt3a in delivery into the brain via the intranasal route mediated anti-apoptotic effects during the early brain injury phase by increasing BCL2/BAX ratio and decreasing cleaved caspase-3 expression (Ruan et al., 2020). The anti-apoptotic effects of Wnt3a were further evidenced by the reduced density of terminal deoxynucleotidyl transferase dUTP nick end labeling (TUNEL)^+^ neurons (Ruan et al., 2020). The administration of Fzd1 or aldolase C siRNA counteracted the beneficial effects of Wnt3a intranasal delivery (Ruan et al., 2020). These results indicate that Wnt3a exerted its neuroprotective effects by alleviating neuronal apoptosis at the cellular and subcellular levels through canonical Wnt pathway activation (Ruan et al., 2020). Interestingly, it has been reported that the number of apoptotic cells positively correlated with Wnt3a and β-catenin mRNA expression, whereas the proliferating cell nuclear antigen (PCNA)^+^ cells negatively correlated with Wnt3a and β-catenin mRNAs during the early brain injury phase and the second phase of hemorrhagic stroke (Zhou L. et al., 2014). Collectively these findings suggest that the canonical Wnt pathway regulate the subtle balance between cell apoptosis and survival within the damaged region after hemorrhagic stroke (Zhou L. et al., 2014).

Using the potent cell-penetrating peptide D-JNKI-1, which selectively blocks JNK and c-Jun interaction, it has been shown that deactivation of the JNK/c-Jun pathway significantly decreased the lesion volume, hemispheric swelling and improved the neurological functions after ICH, correlating with an increased expression of aquaporin-4 (AQP4) in astrocyte endfeet (Michel-Monigadon et al., 2010). Iron contributes to ICH-induced brain injury (Xi et al., 2006), as free iron facilitates free radical formation and oxidative brain damage via activation of the JNK/c-Jun pathway (Wan et al., 2009). Administration of deferoxamine (DFX), an iron chelator, decreased the level of p-JNK in the basal ganglia of rats after SAH during the early brain injury phase, reduced free iron contents in the cerebral spinal fluid (CSF), and improved neurological recovery (Wan et al., 2009). In addition, administration of SP600125, a JNK inhibitor, in rats after SAH increased the density of NeuN^+^ cells within the periphery of the hematoma, without affecting the hematoma size and brain water content (Ohnishi et al., 2007). Interestingly, attenuation of JNK activation via SP600125 administration increased microglial cell death (Ohnishi et al., 2007), and reduced the pro-inflammatory response following SAH notably by decreasing the levels of IL6 in the CSF during the early brain injury phase as well as the second phase of hemorrhagic stroke in dogs (Yatsushige et al., 2005). In another study, JNK inhibition using SP600125 has been shown to reduce the levels of JNK and c-Jun phosphorylation, correlating with an attenuation of the angiographic and morphological vasospasm of the basilar artery in the brain of dogs after SAH (Yatsushige et al., 2005, 2008). Furthermore, SP600125 administration significantly attenuated the infiltration of leukocytes, namely T cells, neutrophils, and macrophages, associated to a reduced apoptosis (Yatsushige et al., 2005, 2008). The exogenous intranasal injection of recombinant Dkk3 (rDkk3), also called SRP6268, decreased JNK phosphorylation and AP1 expression, reduced the brain water content during the early brain injury phase, and improved long-term neurological functions after ICH (Xu et al., 2020). In addition, rDkk3 exerted anti-inflammatory effects within the peri-hematomal zone after ICH induction translated by a reduced expression of TNFα and IL1β (Xu et al., 2020). Interestingly, these beneficial effects were abolished following the injection of Kremen1 or Dvl1 siRNAs (Xu et al., 2020). The early administration of JNK1 siRNA improved the neurological recovery and survival rate after SAH, indicating that interruption of the early brain injury process support neuronal survival in the subsequent phases of SAH (Ling et al., 2019). Indeed, JNK1 siRNA attenuated neuronal apoptosis by decreasing p53 phosphorylation as well as mitochondrial apoptotic pathways via the downregulation of BAX, upregulation of BCL2, and downregulation of cleaved-caspase-3, thus preserving neurons from undergoing apoptosis after SAH (Ling et al., 2019). These findings suggest that deactivation of the non-canonical Wnt pathway provides protection in the context of hemorrhagic stroke. Indeed, it has been shown that the RHOA/ROCK pathway was also activated after hemorrhagic stroke. Recent clinical studies have reported a slight improvement in the clinical outcomes of patients treated with the ROCK inhibitor fasudil 24 h following hemorrhagic stroke onset (Zhao et al., 2011, 2006). In experimental studies, administration of fasudil or ROCK2 specific inhibitor KD025, did not affect the neurological outcome or the size of hematoma after ICH (Akhter et al., 2018). It is noteworthy to mention that KD025 was less potent than fasudil in stopping intracerebral bleeding, whereas it was more potent in reducing the hematoma size (Akhter et al., 2018). In another study, fasudil alone did not inhibit the RHOA activity, whereas pitavastatin attenuated RHOA activation in VSMCs following SAH (Naraoka et al., 2013). The combination of fasudil and pitavastatin strongly reduced RHOA activity in VSMCs, and improved the cross-sectional area of basilar artery after SAH (Naraoka et al., 2013). Statins have already been shown to prevent vasospasm via induction of eNOS (Sabri et al., 2011). Pitavastatin alone or in combination with fasudil significantly increased eNOS release, thus preventing cerebral vasospasm, an effect that was absent in animals treated only with fasudil (Wright et al., 1990). The administration of Y-27632, a ROCK inhibitor, attenuated brain injury and ameliorated neurological functions in the early brain injury phase after ICH, which was accompanied with an increased expression of the adherens junction proteins (Huang et al., 2012). However, another report showed that Y-27632 reduced brain edema after SAH without affecting neurological outcomes (Fujii et al., 2012). In this study, the authors have compared the effects of Y-27632 with hydrofasudil, and proposed that hydrofasudil is more potent than Y-27632 in SAH, as it improved neurological recovery (Fujii et al., 2012). Furthermore, hydrofasudil inhibited ROCK activity, which correlated with a reduced BBB permeability associated to an increased expression of occludin and ZO1 (Fujii et al., 2012). Implication of RHOA activation in destabilizing endothelial cell junctions and altering BBB integrity following ICH model was validated using the RHOA inhibitor, transferase C3 (Zhao H. et al., 2016). The intraperitoneal injection of transferase C3 reduced BBB permeability by increasing the expression of occludin, ZO1, and VE-cadherin, and improved the neurological functions in rats after ICH (Zhao H. et al., 2016). The P2X7 receptor, which is known for its cytotoxic activity (Volonté et al., 2012), was activated very early within the peri-hematomal zone after ICH (Zhao H. et al., 2016). Exogenous intraperitoneal delivery of A-438079, a competitive antagonist of P2X7, significantly decreased RHOA activation, and alleviated the neurological deficits after ICH (Zhao H. et al., 2016). In addition, P2X7 receptor was detected in endothelial cells and astrocytes, and its deactivation using A-438079 significantly improved BBB integrity by increasing the expression of occludin, ZO1, and VE-cadherin (Zhao H. et al., 2016). Administration of P2X7 siRNA in mice replicated the effects of A-438079, validating the implication of P2X7 (Zhao H. et al., 2016). Interestingly, these beneficial effects were strongly attenuated when A-438079 was administered in combination with BzATP, an agonist of P2X7 receptor (Zhao H. et al., 2016). Another study reported that P2X7 receptor is expressed as well in neurons after ICH (Wen et al., 2017). The administration of BBG, a P2X7 antagonist, reduced the expression of phosphorylated p38, extracellular signal-regulated kinases (ERKs), and NF-κB; however, expression and phosphorylation of JNK and c-Jun remained unchanged (Wen et al., 2017), suggesting that P2X7 receptor activation modulated essentially RHOA activity. The administration of BBG decreased brain edema as well as the number of Fluoro-Jade (FJB)^+^ cells, and reduced the level of cleaved-caspase-3 (Wen et al., 2017).

The recent findings are suggesting that CaMKII activity, another main component of the non-canonical pathway, might be implicated in artery contraction or vasospasm (House et al., 2008), associated to poor prognosis after hemorrhagic stroke. Indeed, CaMKII expression was reported to increase in the arteries after SAH (Edvinsson et al., 2014). The administration of KN93 attenuated the SAH-induced contractions mediated by endothelin-1 (ET1) and 5-hydroxytryptamine (5-HT), a contractile protein implicated in basilar and MCA arteries, and ameliorated the sensorimotor function of rodents after SAH (Edvinsson et al., 2014). Another report has demonstrated that CaMKII phosphorylation is highly expressed in neurons and microglia in the brain of rodents after SAH (Zhou et al., 2018). The intraperitoneal injection of dihydrolipoic acid (DHLA), an active form of the lipoic acid (LA), reduced CaMKII and JNK activities, and improved the short- and long-term neurological recovery after SAH (Zhou et al., 2018). DHLA administration promoted as well the protective anti-inflammatory phenotype of microglia (Zhou et al., 2018) (Supplementary Table 2).

## The Wnt Pathway in TBI

TBI is defined as a brain damage resulting from an external mechanical force, leading to temporary or permanent impairment in cognitive, physical, and psychosocial functions (Lambert et al., 2016). TBI constitutes the main cause of death and disability in the young adults, and contributes to the increasing costs of health care due to its high incidence rate and often long-term sequelae (Hyder et al., 2007; Najem et al., 2018). However, there is still no effective therapy available, in part due to the poor understanding for the pathobiology of this neurological condition (Hyder et al., 2007). TBI is characterized by a complex pathogenesis comprising primary and secondary injury mechanisms (Galgano et al., 2017). The primary injury is the result of the immediate mechanical disruption of the brain tissue that occurs at the time of exposure to the external force and includes contusion, damage to blood vessels, and axonal disorganization (Galgano et al., 2017). The secondary injury evolves over minutes to months after the primary injury, and is the result of cascades of metabolic, molecular and cellular events that ultimately lead to cell death, tissue damage, and brain atrophy (Xiong et al., 2013; Galgano et al., 2017), as well as altered cognitive functions (Lambert et al., 2016). Activation of astrocytes and microglia constitutes an important pathophysiological process after TBI. Indeed, astrocytes are recruited to the lesion site and are quickly activated in response to injury after TBI (Karve et al., 2016). Activated astrocytes have been shown to play an important role in neuroprotection by releasing various trophic factors after TBI (Karve et al., 2016). Autophagy is activated under stress conditions to maintain cell survival by allowing the recycling of macromolecules and metabolites for new protein synthesis modulating (Zhang and Wang, 2018). Autophagy has been shown to be dysfunctional in TBI. Indeed, inhibition of autophagy following TBI reduced cell loss and lesion volume, as well as ameliorated neurological functions (Lipinski et al., 2015; Zhang and Wang, 2018). Importantly, β-catenin negatively regulates autophagy via direct inhibition of autophagosome formation (Zhang et al., 2018). LC3, a key autophagosomal component that is usually upregulated during autophagy, has been shown to form a complex with β-catenin for autolysosomal degradation (Zhang et al., 2018). Moreover, TBI constitutes a major risk factor for AD. Indeed, amyloid-β (Aβ) deposition, which is one of the pathological hallmarks of AD significantly increases following TBI in animal models as well as in humans (Yu et al., 2012b). During the last decade, the overwhelming emerging findings are suggesting that the Wnt pathway play important roles in TBI pathobiology, thereby constituting a novel target for the development of novel therapeutic interventions.

### Implication in TBI Pathobiology

Investigations into the implication of the Wnt pathway in humans with TBI conditions have recently started. In a recent study, it was reported that the levels of Dkk1 were elevated in the serum of patients with severe TBI (Ke et al., 2020). Importantly, Dkk1 elevated levels in the serum were closely associated with increasing severity of the trauma and higher risk of short-term mortality (Ke et al., 2020). Furthermore another study has reported that RHOA was upregulated as early as 24 h after trauma, persisting for several months after TBI (Brabeck et al., 2004). In this study, RHOA was upregulated in various cell types including granulocytes, monocytes, macrophages, as well as reactive astrocytes, and to a lesser extent in neurons (Brabeck et al., 2004).

**FIGURE 3:**
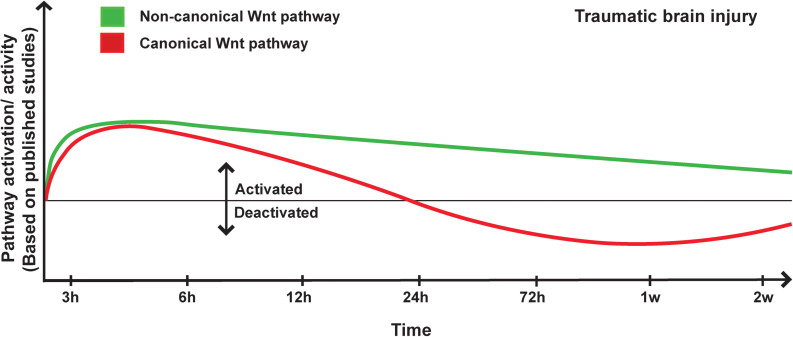
Proposed scheme for Wnt pathway temporal in traumatic brain injury. A representation of canonical and non-canonical Wnt pathways temporal regulation following traumatic brain injury (TBI) onset based on pathway activation (i.e., modulation of pathway’s key components) and activity (i.e., regulation of specific target genes), as reported in the different published studies.

In the recent years, we began to get better insights into the involvement of Wnt pathway in TBI from experimental studies using different TBI animal models. Most of these studies emphasized on the role of β-catenin, the main effector of the canonical Wnt pathway. Indeed, it has been shown that β-catenin expression was increased in the peri-lesional microvasculature of mice at 1 and 7 days post-TBI, whereas it was reduced in injury site 7 days post-TBI (Salehi et al., 2018). A similar expression pattern was reported in a transgenic mouse line reporter for the canonical Wnt pathway activity, TCF:LEF1:H2B-GFP mice (Salehi et al., 2018). Indeed, β-catenin expression increased 24 h after TBI, and was associated to an enhanced angiogenesis within the peri-lesional zone (Salehi et al., 2018). Importantly, the density and complexity of the microvasculature increased at day 7 after TBI (Jullienne et al., 2018). The mRNA levels of β-catenin and Wnt3a significantly increased following TBI, peaking at day 3 post-TBI for β-catenin and at day 7 for Wnt3a (Wei et al., 2020). Furthermore, several findings have demonstrated that Akt/GSK3β pathway regulates as well β-catenin expression (Zhao et al., 2012). Phosphorylated Akt (p-Akt, i.e., activated) deactivates the kinase activity of GSK3β kinase activity via phosphorylation on serine (Zhao et al., 2012). In the injured cortex of rats, p-Akt increased 4 h after impact, decreasing 72 h later, and was accompanied by increased levels of p-serine-GSK3β 4 h after impact, peaking 72 h later after TBI (Zhao et al., 2012). P-Akt-mediated GSK3β kinase inhibition promoted β-catenin stabilization and accumulation as early as 4 h after the impact and remained constant for 7 days (Zhao et al., 2012). Similar findings were reported in another independent study, which showed in addition that TBI induced a rapid transient increase in LRP6 phosphorylation followed by a slight decrease in β-catenin phosphorylation mediated by GSK3β (Dash et al., 2011). Evidence is suggesting that activated astrocytes limit damage expansion, stimulate tissue repair, and promote synaptic remodeling after TBI (Burda et al., 2016). Using a transgenic mouse line reporter for β-catenin transcriptional activity, BATGAL mice, it has been demonstrated that β-catenin signaling was associated to an enhanced proliferation of neural/glial antigen-2 (NG2)^+^ progenitors and reactive astrocytes after TBI (White et al., 2010). Moreover, the expression of DIX domain-containing protein-1 (DIXDC1), a positive regulator of the canonical Wnt pathway that activates Wnt3a signaling through Dvl2, was up-regulated in neurons and astrocytes following TBI, and was implicated in reactive astrocyte proliferation (Lu et al., 2017). Interestingly, expression of the C-terminal-binding protein-2 (CtBP2), a protein involved in the transcriptional regulation of Wnts, was induced at the peri-lesional zone after TBI (Zou et al., 2013). CtBP2 was highly expressed in proliferating astrocytes, and was associated to an increased expression of BCL2 (Zou et al., 2013). TBI stressors have been shown to trigger the intracellular accumulation of Ca^2+^ and cyclic adenosine monophosphate (cAMP), activating PKC, CaMKII and protein kinase-A (PKA), which in turn phosphorylate the cAMP response element binding factor (CREB) and serum response factor (SRF), subsequently increasing c-Jun transcriptional activity and cell death (Sheng and Greenberg, 1990; Morgan and Curran, 1991; Raghupathi et al., 2000; Raghupathi, 2004). Interestingly, an increased mRNA expression of c-Jun was observed 5 min after TBI and was maximal 30 min later, persisting for 6 h post-TBI (Raghupathi et al., 1995). Modulation of other components of the non-canonical Wnt pathway was reported after TBI. For instance, CAMKIIδ expression increased within the areas surrounding the impact core, peaking 3 days later and then returned to normal levels (Pan et al., 2014). CAMKIIα plays a key role in regulating the formation of hippocampal-dependent memory, and its activity was reported to increase in the CA1, CA3 and DG regions as early as 30 min after trauma (Atkins et al., 2006; Folkerts et al., 2007). Importantly, CAMKIIα increased activity was accompanied by the phosphorylation and activation of the AMPA-type glutamate receptor (GluR1), and the cytoplasmic polyadenylation element-binding protein (CPEB) in the hippocampus and cortex 1 h after TBI (Atkins et al., 2006). These findings suggest that the biochemical cascades implicated in memory formation are activated in non-selective manner in neurons after TBI (Atkins et al., 2006). However, a proper memory formation requires activation of the CAMKIIα signaling in specific neuronal synapses, and the non-selective activation of this signaling in all synapses may disrupt memory formation, which may account for the memory loss after TBI (Atkins et al., 2006). The recent findings have demonstrated that the RHOA/ROCK pathway is regulated after TBI. RHOA activation was observed from 24 h until 3 days after impact in the cortex, and at 3 days in the hippocampus of the brain of a rat following TBI (Dubreuil et al., 2006). In CamKIIα-Cre;RHOA^fl/fl^ mice, RHOA cKO mice, in which RHOA was specifically depleted in postnatal neurons, motor and cognitive functions were persevered 14 days after TBI without substantially influencing the lesion volume (Mulherkar et al., 2017). These studies highlight an important role of the Wnt pathway in TBI pathobiology, but more research is still needed to fill-in the exiting gaps in the literature (Figure 3).

### Implication in TBI Therapy

Similar to ischemic stroke, in response to TBI, the brain tries to repair itself by regulating neurogenesis, angiogenesis, and inflammation (Xiong et al., 2010; Russo and McGavern, 2016). After TBI, neurogenesis is stimulated in the SGZ of the DG of the hippocampus and in the SVZ to replace the cells lost by apoptosis or necrosis caused by the impact (Dash et al., 2001; Rice et al., 2003; Sun et al., 2005). Indeed, TBI has been shown to promote the migration of progenitors from the SVZ to the injured site (Ramaswamy et al., 2005). In a rat model of TBI, cell proliferation was reported in the SVZ, corpus callosum, around the cortex and sub-cortical areas connected to the injured site but not inside the lesion site (Kernie et al., 2001). These newborn cells originating from the SVZ were shown to differentiate into neurons or glial cells (Kernie et al., 2001). Cognitive recovery has been observed 2 weeks after TBI, strongly correlating with the integration of newborn cells into the pre-existing network (Emery et al., 2005; Urrea et al., 2007). TBI occasions a serious damage to the microvasculature, which has been shown to occur in both hemispheres (Obenaus et al., 2017). Cerebral hypoperfusion, hypoxia, ischemia, BBB disruption, hemorrhage, and edema formation constitute the main consequences of microvascular dysfunction associated to TBI (Salehi et al., 2017). Interestingly, the endogenous microvascular repair processes were shown to occur in a time course of 2 to 3 weeks starting 2 days post-trauma (Morgan et al., 2007; Beck and Plate, 2009). Importantly, the newly formed capillaries seem to originate from the peri-lesional tissue to penetrate inside the lesion core (Sköld et al., 2005; Park et al., 2009; Hayward et al., 2011). An increased frequency of endothelial progenitor cells (EPCs) has been reported in the blood circulation following TBI, as well as in the microvasculature located inside the lesion site, outlining an active angiogenic response (Guo et al., 2009; Gong et al., 2011; Liu L. et al., 2011). TBI also triggers a strong inflammation response that has been shown, if remained uncontrolled to underlie the chronic neurodegeneration associated to TBI (Johnson et al., 2013; Smith et al., 2013; Faden and Loane, 2015). The resident and peripheral immune cells quickly respond to brain injury and participate to the repair process as well after TBI (Das et al., 2012; Finnie, 2013). Microglia and astrocytes are among the first cells to respond and actively contribute in supporting the survival of stressed neurons, removing of cell debris, releasing trophic mediators, and participating in astroglial scar formation (Corps et al., 2015; Zhou et al., 2020). As Wnt pathway activation after TBI actively contribute in regulating neurogenesis, angiogenesis, and inflammation, research in the field is attempting to anticipate in targeting the pathway in order to promote structural and functional repair after trauma (Marchetti and Pluchino, 2013; Wu et al., 2013; Zhang et al., 2013).

Similar to ischemic and hemorrhagic stroke, several studies were interested in assessing the effects of Wnt ligands in developing TBI therapies, with an emphasis on Wnt3a. When delivered intranasally after TBI, Wnt3a rescued motor function and enhanced the number of NeuN^+^ cells without affecting the reactivity of astrocytes (Zhang et al., 2018; Chang et al., 2020). Wnt3a exogenous administration efficaciously activated the canonical Wnt pathway by increasing β-catenin nuclear levels, promoted neurogenesis and reduced lesion volume (Zhang et al., 2018; Chang et al., 2020). Similar results were obtained with the intravenous injection of recombinant Wnt3a as well as the intravenous injection of mesenchymal stem cells (MSCs), which enhanced hippocampal neurogenesis by stimulating Wnt3a release after TBI (Zhao Y. et al., 2016). The administration of simvastatin for 2 weeks following TBI increased the expression of p-PKB, CREB, BDNF, and VEGF in the DG (Wu et al., 2008). The increased expression of these factors positively correlated with NPCs proliferation and differentiation into mature neurons in the DG, significantly attenuating spatial learning deficits (Wu et al., 2008). These effects were mediated by canonical Wnt pathway activation associated to the inhibitory effects of simvastatin on GSK3β activity (Wu et al., 2008). Furthermore, simvastatin therapy for 14 days after TBI reduced axonal injury, enhanced neurite outgrowth, and ameliorated neurological recovery (Wu H. et al., 2012). Interestingly, membrane depolarization plays a decisive role in mediating the survival and maturation of newborn neurons in the DG (Zhao et al., 2018). Optogenetic tools were applied to stimulate the membrane depolarization of Dcx^+^ cells via the injection of LV-Dcx-channelrhodopsin-2 (ChR2)-EGFP gene into the brain of TBI mice (Zhao et al., 2018). Optical depolarization of Dcx-EGFP^+^ cells between 3 and 12 days after TBI attenuated cognitive deficits, accompanied by an enhanced survival and maturation of the newly generated cells in the DG (Zhao et al., 2018). Importantly, these effects were abolished upon Dkk1 administration, confirming that the survival and maturation or newborn neurons were essentially mediated via canonical Wnt pathway activation (Zhao et al., 2018). The intranasal administration of Wnt3a has been shown as well to increase GDNF and VEGF expression, subsequently enhancing angiogenesis after TBI (Zhang et al., 2018). Furthermore, the intranasal administration of Wnt3a reduced cell death and improved functional recovery after TBI (Zhang et al., 2018). Additionally, lithium application for 5 days after TBI improved learning and memory capabilities of animals, associated to a reduced neuronal loss in the CA3 region of the hippocampus (Dash et al., 2011).

Activation of the canonical Wnt pathway via the pharmacological inhibition of GSK3β to stabilize β-catenin after TBI efficaciously reduced cell death, improved locomotor coordination, attenuated depression and anxiety behaviors, and ameliorated overall cognitive functions (Yu et al., 2012a; Shim and Stutzmann, 2016). Indeed, lithium administration has been reported to attenuate BBB disruption following TBI (Yu et al., 2012a). Administration of lithium 15 min after cortical contusion injury (CCI) followed by daily administration for 3 to 6 h or 2 weeks after CCI or once daily for 3 days reduced the inflammatory responses translated by attenuated activation of microglial cells and MMP9 expression (Yu et al., 2012a). This was associated to a reduced lesion size and improved of cognitive functions (Yu et al., 2012a). The authors proposed that injection of lithium within 3 h after TBI provide the best neuroprotective and anti-inflammatory effects (Yu et al., 2012a). Furthermore, a single acute injection of valproic acid (VPA), a potent GSK3β inhibitor, strongly reduced BBB breakdown and diminished the lesion volume after CCI. When injected 5 days after TBI, VPA improved motor function, spatial learning and memory (Dash et al., 2010). Interestingly, the beneficial effects of VPA were time and dose dependent, outlining its potency in activating the canonical Wnt pathway (Dash et al., 2010). As such, the authors suggested that an optimal beneficial effect on structural and functional recovery could be achieved with a first acute injection applied 30 min after TBI followed by daily administration of VPA for at least 5 days (Dash et al., 2010). Since lithium and VPA exhibited beneficial effects in TBI, a co-treatment with sub-effective doses was evaluated. The co-treatment reduced brain lesion, BBB disruption and drastically improved long-term functional recovery (Yu et al., 2013). The authors suggested that a regimen that comprises sub-effective doses might reduce side effects and increase the tolerance (Yu et al., 2013). The prolonged application of VPA after TBI for 3 weeks reduced dendritic loss in the hippocampus (Dash et al., 2010). Similar to lithium, simvastatin and VPA, resveratrol, which is an antioxidant, has been shown to possess inhibitory effects against GSK3β (Lin et al., 2014). Resveratrol mitigated cell death in an *in vitro* model of TBI by suppressing essentially GSK3β-mediated reactive oxygen species (ROS) generation (Lin et al., 2014). Moreover, the co-treatment of lithium and VPA attenuated neurodegeneration after TBI (Yu et al., 2013). Although more investigations are required, the accumulating findings are indicating that GSK3β inhibitors constitute indeed promising candidates for TBI treatment. TBI is a condition that substantially increases the risk of developing dementia-like pathologies, such as AD. For instance, lithium administration 15 min then once daily for up to 3 weeks after TBI attenuated Aβ accumulation, increased beta-site amyloid precursor protein (APP)-cleaving enzyme-1 (BACE1) expression in the hippocampus and the corpus callosum, accompanied by reduced hyper-phosphorylation of tau in the thalamus (Yu et al., 2012b). Application of lithium for 20 days ameliorated short-term memory and learning (Yu et al., 2012b).

Various non-chemical treatments have also been tested to further activate the Wnt pathway after TBI for therapeutic purposes. For instance, acupuncture in rats with TBI induced the mRNA and protein expression of Wnt3a, β-catenin and sex determining region Y-box 2 (SOX2), a transcription factor implicated in the maintenance of NSCs (Zhang et al., 2016). Indeed, this was associated to enhanced proliferation and differentiation of NPCs (Zhang et al., 2016). Interestingly, it has been shown that hyperbaric therapy could restore oxygen supply after TBI, thereby increasing the expression of various antioxidant genes that alleviate inflammation and apoptosis, while promoting neurogenesis, and angiogenesis (Thom, 2009; Godman et al., 2010; Liu W. et al., 2011). Mice in which TBI was induced were placed in a hyperbaric chamber for 90 min. This regimen reduced the number of apoptotic neurons, as well as the mRNA expression of caspase-3 and the level of cleaved-caspase-3 (He et al., 2019). These effects were associated to an increased expression of β-catenin and decreased expression of GSK3β (He et al., 2019).

The non-canonical pathway has also been shown to constitute an interesting therapeutic target for TBI. Fasudil, a RHOA/ROCK inhibitor, as well as RHA depletion prevented TBI-induced spine remodeling and mature spine loss in hippocampal pyramidal neurons, thus improving neurological recovery (Mulherkar et al., 2017). Moreover, docosahexaenoic acid (DHA), which has been shown to inhibit JNK, rescued TBI-mediated hippocampal long-term potentiation (LTP) and improved hippocampus-dependent learning and memory as well as enhanced motor function (Zhu W. et al., 2019). These results are in line with previous studies demonstrating that neuronal dysfunction was alleviated by DHA (Zhu et al., 2017, 2018). A natural supplementation of blueberry for 2 weeks showed mitigated loss of spatial learning and memory performances and improved anxiety (Krishna et al., 2019), associated to increased expression of BDNF, which plays crucial role in neural maturation, and activated CAMKII (Krishna et al., 2019). It is noteworthy to mention that hemorrhage often occurs after TBI due to BBB breakdown. Basic fibroblast growth factor (bFGF) administration reduced RHOA activity and increased the expression of tight junction proteins, thus preserving BBB integrity after injury (Wang Z.-G. et al., 2016). bFGF increased the expression of claudin-5, occludin, ZO1, and β-catenin in human brain microvascular endothelial cells (HBMECs) exposed to oxygen glucose deprivation (OGD)/re-oxygenation to mimic TBI conditions (Wang Z.-G. et al., 2016). Inhibition of PI3K/Akt pathway or RAC1, using LY-294002 or RAC1 siRNA respectively, abolished the protective effects of bFGF on BBB integrity (Wang Z.-G. et al., 2016). The administration of saikosaponins, which are triterpene saponins isolated from *Bupleurum*, decreased the expression AQP4 expression that is implicated in swelling, MMP9, mitogen-activated protein kinase (MAPK), JNK, TNFα, and IL6 (Mao et al., 2016). Saikosaponins reduced brain edema, BBB breakdown, and inflammation, as well as ameliorated neurological recovery (Mao et al., 2016). Administration of the JNK inhibitor, SP600125, for 7 days after TBI in mice decreased the expression of NF-kB, which in turn reduced caspase3 expression in neurons (Rehman et al., 2018; Bhowmick et al., 2019). On the other hand, ROCK inhibition after TBI in mice promoted acute neuroprotection and functional recovery with only modest impact on neurogenesis, astrocytic reactivity and macrophage/microglial activation (Bye et al., 2016). TBI triggers a cascade of events that increase the concentration of intracellular Ca^2+^, which is further exacerbated by the increased expression of Wnt5a and Fzd2, a major component of the non-canonical Wnt pathway (Niu et al., 2012). Inhibition of Wnt5a/Fzd2 interaction using Box5, a Wnt5a-derived hexapeptide that antagonizes Wnt5a-mediated cellular activities or Wnt5a siRNA, could constitute a potential therapy to alleviate Ca^2+^ increase and protect neurons (Niu et al., 2012). As mentioned earlier, TBI constitutes a high-risk factor for developing dementia. DHA administration deceased JNK expression, subsequently reducing TBI-mediated tau phosphorylation (Zhu W. et al., 2019). Collectively, these studies suggest that Wnt pathway constitute an important target for the development of efficacious protective and restorative therapies for TBI (Supplementary Table 3).

## Conclusion

**FIGURE 4:**
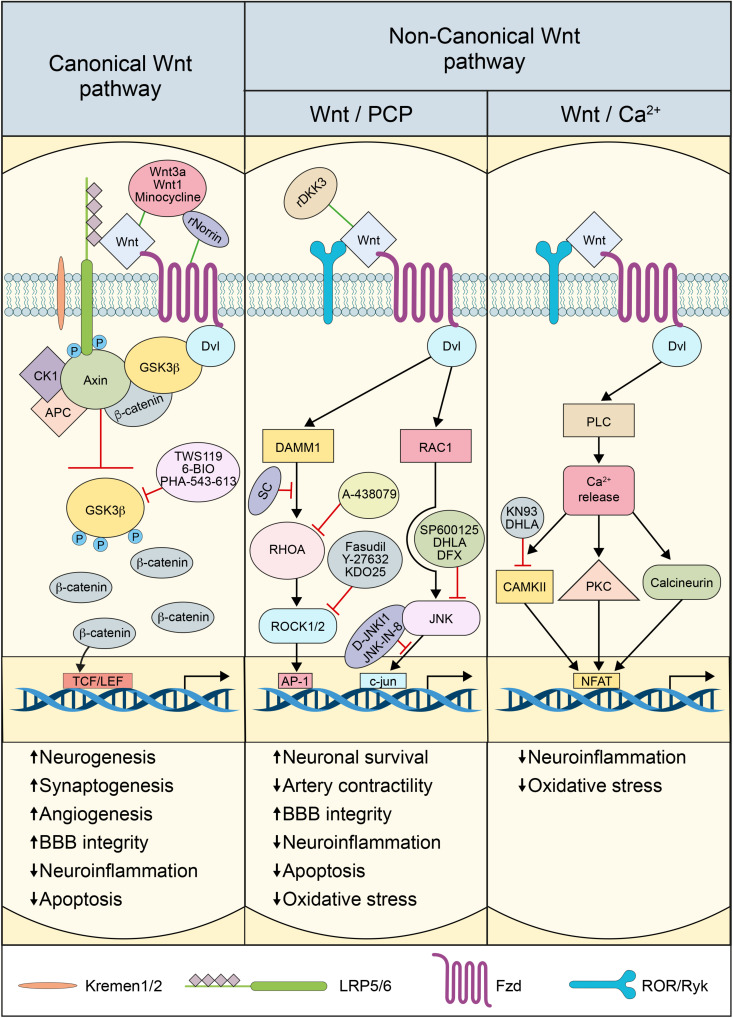
Scheme depicting Wnt pathway modulation for therapeutic purposes: An illustration summarizing the key approaches applied in the literature to modulate canonical and non-canonical Wnt pathways activation and activity, as a strategy to attenuate brain injury, and to ameliorate the post-injury reparative and restorative process.

The mechanisms underlying brain injury in ischemic and hemorrhagic stroke as well as TBI are very complex and multiphasic, thus constituting a major challenge in the development of efficacious therapeutic interventions. Despite efforts, still no disease-modifying therapy exit for these neurological conditions. This void is frustrating as the emerging findings are indicating that the injured brain tissue is not just passively dying over time, but it is actively trying to recover. Indeed, various developmental and ontogenic processes have been shown to re-emerge upon injury, such as neurogenesis, neural plasticity, angiogenesis, gliosis, and others (Lo, 2008; Hermann and ElAli, 2012; Addington et al., 2015). These processes clearly translate an attempt from the brain to self-repair and regenerate, thus providing a new framework for the development of novel therapeutic interventions. Yet the major challenge remains in identifying a “drugable” target that could be modulated to fine-tune the injury-induced developmental and ontogenic processes. The Wnt pathway regulates crucial biological aspects throughout lifespan. It is critically implicated in regulating the intimate emergence and patterning of the nervous and vascular systems (Hermann and ElAli, 2012; Noelanders and Vleminckx, 2017). The experimental findings are convincingly indicating that the canonical Wnt pathway is of particular interest (Figure 4). Indeed, genetic alteration of the pathway drastically impact injury progression, repair and regeneration. Furthermore, pathway activation seems to potently stimulate injured tissue protection and restoration as well as ameliorating already FDA approved therapies, such as thrombolysis via rtPA for ischemic stroke (Jean LeBlanc et al., 2019). Data from patients are indicating that some component of the pathway could be even used as biomarkers or prognostic tools, such as Dkk1 (Seifert-Held et al., 2011; Zhu Z. et al., 2019). The canonical Wnt pathway is an attractive target from pharmacological point of view, as several modulators have been developed in the past decades for other medical conditions, namely neurodegenerative disorders and cancer. The accumulating evidence is suggesting that in order to achieve maximal effects, the implication of some endogenous inhibitors such as Scl and Dkk1 should be taken into consideration. Indeed, the elevated endogenous expression Dkk1 within the injured tissue was sufficient to abolish the biological activity of potent ligands such as Wnt3a (Wei et al., 2018). Dkk1 expression is regulated in an age- and biological sex-dependent manner, thus offering new directions in developing tailored Wnt-dependent therapeutic interventions (Zhang et al., 2008; Seib et al., 2013). On the other hand, the complexity of non-canonical Wnt pathway’s intracellular signaling makes it difficult to fully appreciate its exact role as several components of the pathway are shared with Wnt-independent pathways. However, the non-canonical Wnt pathway seems to counteract several of the biological effects of the canonical pathway. Nonetheless, ROCK seems to constitute a very promising target for the therapeutic purposes. It is clear that more research is still needed to fully elucidate with some specificity the implication of the non-canonical Wnt pathway in brain injury and repair.

## Author Contributions

RM contributed to the writing and figure preparation. SL contributed to the writing. AE contributed to the writing, editing, and finalization of the manuscript. All authors contributed to the article and approved the submitted version.

## Conflict of Interest

The authors declare that the research was conducted in the absence of any commercial or financial relationships that could be construed as a potential conflict of interest.
