# Supplementary material for: Wnt Pathway: An Emerging Player in Vascular and Traumatic Mediated Brain Injuries
Source: Front Physiol. 2020 Sep 18;11:565667. doi: 10.3389/fphys.2020.565667 (PMC7530281; doi:10.3389/fphys.2020.565667)
Supplement: Supplementary file 1 [file Table_1.DOCX]

| **Studies** | **Species** | **Stroke type/ Experimental models** | **Treatment** | **Route of delivery** | **Time of delivery** | **Biological processes** | **Main effects**  **(Key markers used)** |
| --- | --- | --- | --- | --- | --- | --- | --- |
| **Canonical and non-canonical Wnt pathway - Clinical findings** | | | | | | | |
| Seifert-Held et al. (2011) | Human | Acute ischemic stroke | N/A | N/A | N/A |  | - Increased level of Dkk1 in plasma  - No significant differences of Dkk1 in subtypes of ischemic stroke  - No correlation of Dkk1 levels was found with stroke severity at day 90 |
| He et al. (2016) | Human | - Large artery atherosclerotic stroke  - Small artery occlusion stroke | N/A | N/A | N/A |  | - Increased level of Dkk1 and Scl in serum  - No difference was detected between the stroke subtypes and stroke severity |
| Zhu et al. (2019) | Human | Acute ischemic stroke | N/A | N/A | N/A |  | - Increased level of Dkk1  - Elevated Dkk1 levels were associated with an increased risk of primary outcome  - High serum Dkk1 levels were associated with poor prognosis at 1 year |
| Harriott et al. (2015) | Human | Different sub-types of ischemic stroke:  - cardioembolic  - large vessel  - small vessel  - others  - undetermined | N/A | N/A | N/A |  | - LRP6 and LRP1 variants may be associated with risk of ischemic stroke |
| Sun et al. (2020) | Human | Cerebral infarction | N/A | N/A | N/A |  | - Increased level of miR-150-5p in plasma |
| Shibuya et al. (2005) | Human | Acute ischemic stroke | Fasudil,  (60mg/60min/14days) | Intravenous | Within 48 hours after onset |  | - Improved the patient's clinical outcome |
| Feske et al. (2009) | Human | Acute ischemic stroke | Fasudil  (1μM) | N/A | N/A |  | - Increased the ROCK activity at 48 hours.  Fasudil :  - Decreased ROCK activity of polymorphonuclear leukocytes from blood samples *in vitro* |
| **Canonical Wnt pathway - Experimental findings** | | | | | | | |
| Kelly et al. (2004) | Male Sprague–Dawley rats | Transient MCAo  (90 minutes) | 1) Chir025  (25 mg/kg)  2) Chir025  (15mg/kg) | Intravenous | 1) Immediately on onset  2) 1.5 hours, 9.5 hours after onset and every 12 hours until sacrifice | - Neuronal | Chir025 :  - Decreased infarct volume  - No apoptotic effect (TUNEL+, CC3, CC9)  - Increased level of BCL2 |
| Zhang et al. (2008) | Female Sprague-Dawley rats | Bilateral ovariectomie 1 week before global cerebral ischemia | 1) 17β-estradiol (0.025mg/21days)  2) rDkk1  (1μg/μl/5μl)  3) SP600125  (10mg/kg) | 1) Subcutaneous  2) Intracerebroventricular  3) Tail veins | 1) 1 weeks before onset  2) 12 hours after onset  3) 15 minutes before onset | - Neuronal | 17β-estradiol :  - Attenuated level of Dkk1 in hippocampus CA1 region at 24 hours  - Increased level of Wnt3 and β-catenin at 24 and 48 hours in hippocampus CA1 region  - Decreased level of p-JNK and p-c-jun from 30 minutes to 48 hours  - Decreased neuronal damage cell death at 1week (NeuN+)  - Anti-apoptotic effect (Survivin)  rDkk1 :  - Inhibited the beneficial effect of 17β-estradiol at 1week  SP600125 :  - Attenuated hippocampal CA1 neuronal cell death at 1weeks (NeuN+) |
| Mastroiacovo et al. (2009) | 1) Male Wistar Rat  2) Male C3H and Dblr mice | 1) Transient focal ischemia  2) Permanent focal ischemia | 1) LiCl  (1mg/kg/10days) | Intraperitoneal | 7 days before onset | - Neuronal | - Increased level of Dkk1 in the striatum at 24 hours  - Increased level of Dkk1 in the core and penumbra at day 3  LiCl:  - Decreased reduction of β-catenin  - Did not affect Dkk1 induction in the penumbra  -Decreased infarct volume  Dblr mice :  - Decreased reduction of β-catenin,  - Decreased infarct volume  - Anti-apoptotic affect (BCL2) |
| Chong et al. (2010) | Male Sprague-Dawley rats | Transient MCAo | Wnt1  (24ug/kg)  Wnt1 antibody (15µg/ml)  Dkk1 (24ug/kg) | Internal carotid artery | 30 minutes prior onset and immediately onset |  | - Increased level of Wnt1 from 1 hours to 24 hours in the ipsilateral brain  Wnt 1:  - Decreased infarct volume  - Ameliorated neurological score  Wnt1 antibody and Dkk1  - Increased the infarct volume  - Aggravated the neurologic score |
| Zhou et al. (2011) | Male Sprague–Dawley rats | Permanent MCAo | SB216763 (1.5mg/kg) | Intraperitoneal | Immediately on onset | - Inflammatory | SB216763 :  - Anti-inflammatory effect (IL1β, TNFβ, iNOS)  - Increased autophagy (LC3-II) |
| Xing et al. (2012) | Male Sprague-Dawley rats | Permanent MCAo | Sulindac  (4, 10, or 20 mg/kg) | Intragastric | 30 minuyes before onset | - Neuronal  - Vascular | Sulindac :  - Increased level of β-catenin and Dvl  -Decreased level of p-β-catenin and APC  - Ameliorated functional recovery  - Decreased brain oedema  - Preserved BBB integrity (claudin-5)  - Anti-apoptotic effect (BCL2, BAX) |
| Shruster et al. (2012) | Male C57BL/6 mice | Focal ischemia | LV-Wnt3a-HA | Intracerebroventricular | 4 days before onset | - Neuronal | LV-WNT3a-HA :  - Ameliorated functional recovery from day 2 to day 28  - Increased proliferation, migration of progenitor cells to neuroblasts at 2 day (DCX+, EdU+  - Increased neurogenesis at day 28 (BrdU+/NeuN+)  - Had a neuroprotective effect (BDNF, TUNEL+) |
| Abe et al. (2013) | Male LRP6+/− mice | Transient MCAo  (40 minutes) | N/A | N/A | N/A | - Inflammatory | LRP6+/− mice :  - Decreased level of p-GSK3β-ser9 at 1 hours  - Increased level of mitochondrial p-GSK3β-Y216 at 6 hours  - Had a larger infarct  - Had more severe motor deficit  - Pro-inflammatory effect (COX2, MCP1, ELAM1, PTGES) |
| Modi et al. (2014) | Male Sprague-Dawley rats | Transient MCAo  (120 minutes) | Sulindac (0.2mg/3day) | Subcutaneous | 2 days before onset | - Neuronal | Sulindac :  - Decreased infarct volume  - Anti-apoptotic effect at 3 and 11 days in the penumbra (BCL2, BAK) |
| Wu et al. (2015) | Male Sprague-Dawley rats | Transient MCAo  (60 minutes) | Galangin  (25, 50 and 100 mg/kg) | Intragastric | 15 minutes before onset | - Vascular | Galandin :  - Decreased the level of p-β-catenin/β-catenin ratio  -Increased the level of p-GSK3β/GSK3β ratio  - Decreased neurological deficit  - Decreased NVU damage (Evans blue, claudin-5, ZO1, MMP9) |
| Wang et al. (2016) | Male Sprague–Dawley rats | Transient MCAo  (240 minutes) | TWS119 (30mg/kg)  rtPA  (10mg/kg) | Intraperitoneal | 4 hours after onset | - Vascular | TWS119 :  -Ameliorated behavior outcome at 24 hours  - Preserved BBB integrity at 24 hours (Evans blue, claudin-3, ZO1)  - Decreased haemorrhagic transformation at 24 hours (Hemoglobin)  - Counteracted the negative effect of rtPA |
| Wang et al. (2017c) | Male Sprague–Dawley rats | Transient MCAo  (210 minutes) | TWS119 (30mg/kg) | Intraperitoneal | 3.5 hours after onset | - Vascular | TWS119 :  - Ameliorated behavior outcome  - Preserved BBB integrity (Evans blue, ZO1) |
| Wang et al. (2017b) | Male C57Bl/6 mice | Focal cortex ischemic stroke model | 6-BIO  (8.5ug/kg/2days) | Intraperitoneal | 3 days after onset | - Neuronal | 6-BIO :  - Ameliorated long-term sensorimotor functional recovery at 7, 14 and 21 days  - Increased cell proliferation and migration in the penumbra (BrdU+/DCX+)  - Increased neurogenesis (BrdU+/NeuN+) |
| Wang et al. (2017a) | Male Sprague–Dawley rats | Transient MCAo  (120 minutes) | Lentivirus miR-148b inhibitor (3.0 × 106  IU in 3μl saline) | Intracerebroventricular | 24 hours after onset | - Neuronal | Lentivirus miR-148b inhibitor :  - Ameliorated neurological functional recovery at 4, 7 and 14 days  - Increased neurogenesis at 14 days (NeuN)  - Mediated the proliferation and differentiation of SVZ NSCs (DCX, NG2, Tuj1) |
| Chang et al. (2017) | Male and emale Gpr124flox- and Gpr124flox/−Ctnnb1lox(ex3)/+;Cdh5-CreER mice | Transient MCAo  (60 minutes) | N/A | N/A | N/A | - Vascular | Gpr124flox- mice :  - Induced BBB breakdown and hemorrhagic transformation (Hematoxylin-eosin ,Perl’s Iron)  Gpr124flox/−Ctnnb1lox(ex3)/+;Cdh5-CreER mice :  - Increased survival rate  - Reduced infarct volume  - Decreased hemorrhagic transformation (Hematoxylin-eosin ,Perl’s Iron)  - Fully restored pericyte coverage around ECs |
| Wei et al. (2018) | Male C57Bl/6 mice | Focal ischemic stroke | 1) Wnt3a  (2ug/kg)  2) Dkk1  (3ug/kg) | Intranasal | 1 hours after onset | - Neuronal  - Vascular | Wnt3a :  - Increased neurogenesis (BrdU+/NeuN+, DCX+/BrdU+)  - Increased angiogenesis (GLUT1+/BrdU+, ColIV+/BrdU+ Blood flow)  - Neuroprotective effect  Dkk1 :  - Inhibited the beneficial effect of Wnt3a |
| Qiu et al. (2018) | Male C57Bl/6 and Wip1−/− mice | Permanent MCAo | SB216763 (4mg/kg) | Intraperitoneal | 48 hours after onset | - Neuronal | Wip1/- mice :  - Decreased the level of Wnt3a and β-catenin  - Increased infarct volume  -Decreased neurogenesis (BrdU+/DCX+)  SB216763 :  - Increased the level of β-catenin  - Decreased neurological score  - Decreased infarct volume  - Increased neurogenesis (BrdU+/DCX+) |
| Matei et al. (2018) | Male Sprague–Dawley rats | 1) Transient MCAo  (120 minutes)  2) Permanent MCAo | Wnt3a  (0.4 or 1.2μg/kg) | Intracerebroventricular | 24 hours before onset | - Neuronal | Wnt3a :  1) - Ameliorated neurobehavioral function  - Decreased infarct volume  - Decreased neuronal damage  - Anti-apoptotic effect (CC3)  2) - Increased neurological function  - Decreased infarct volume |
| Zhan et al. (2019) | Male Wistar rats | Transient global cerebral ischemia | 1) Hypoxia  (8% O2 and 92% N2)  2) Lenti-Dkk1  (1.75μl in 3.25μl of 0.9% saline) | 2) bilateral hippocampal CA1 region | 1) 24 hours after onset for 120 minutes  2) 7 days after onset | - Neuronal | Hypoxia :  - Increased the level of Wnt3a and nuclear β-catenin  - Increased Neurogenesis (NeuN)  - Anti-apoptotic effect in CA1 (Survivin, BCL2, BAX)  Lenti-Dkk1 :  - Reversed the HPC‐induced activation level of nuclear β-catenin and p-GSK3β-ser9  - Reversed the neuroprotection in CA1 |
| Jean LeBlanc et al. (2019) | Male C57Bl/6 mice | Transient MCAo  (30 minutes) | 6-BIO  (8ug/kg)  rtPA  (10ug/kg) | Intraperitoneal | 6 hours after onset | - Vascular | 6-BIO :  - Ameliorated behavior function at 24 hours  - Preserved BBB integrity at 24  Hours (IgG, claudin-3)  - Decreased hemorrhagic transformation at 24 hours (Ruptured microvessels)  - Counteracted the negative effect of rtPA |
| Qiu et al. (2019) | Male C57Bl/6 mice | Permanent MCAo | Gastrodin (100mg/kg/days) | Intraperitoneal | 24 hours after onset | - Neuronal | Gastrodin :  - Increased level of Wnt3a and β-catenin at day 7  - Decreased infarct volume at day 7  - Increased neurogenesis at day 7 (DCX+/BrdU+)  - Anti-apoptotic effect at day 7 (CC3, TUNEL+, BCL2, BAX) |
| Zhang et al., (2019) | Male C57Bl/6 mice | Transient MCAo  (60 minutes) | Wnt3a  (2μg/kg/2days) | Intranasal | 1 hour after onset | - Neuronal  - Inflammatory | Wnt3a :  - Decreased brain edema  - Ameliorated neurological score  - Anti-inflammatory effect (IBA1, CD68+, iNOS, TNFα, CD206)  - Anti-apoptotic effect (TUNEL+)  - Decreased A1 (neurotoxic phenotype) and increased A2  (neuroprotective phenotype) astrocytes in the peri-infarct zone |
| Song et al. (2019) | Male C57Bl/6 mice | Focal ischemic stroke + 1 hour hypoxia | TWS119 (10mg/kg/days) | Intraperitoneal | Immediately on onset | - Neuronal  - Vascular  - Inflammatory | TWS119 :  - Ameliorated neurological function at day 14 and 21  - Ameliorated neural plasticity at day 14 and 21 in the peri-infarct zone (GAP43, PSD95, SIM312, Synaptophysin)  - Increased angiogenesis at day 14 and 21 in the peri-infarct zone (BrdU+/CD31+, CD31+)  - Anti-inflammatory effect at day 14 and 21 in the peri-infarct zone (IBA1, CD16/32, CD206, TNFα, IL10) |
| Wang et al. (2020) | Male ICR mice | Transient MCAo  (90 minutes) | 1) OPCs  (6× 10^5^)  2) XAV939 (40 mg/kg) | 1) Intracerebroventricular  2) Intraperitoneal | 24 hours after onset | - Vascular | OPCs :  - Decreased infarct volume at day 3  - Decreased oedema at day 3  - Ameliorated neurological function at day 3  - Increased level of β-catenin on endothelial cells at day 3  - Preserved BBB integrity at day 3 (claudin-5, Occludin)  XAV939 :  - Inhibited the beneficial effects of OPCs |
| Sun et al. (2020) | Male Sprague–Dawley rats | Cerebral infarction | miR-150-5p  (300μg/3days) | Tail vein | Immediately on onset |  | miR-150-5p :  - Increased level of β-catenin, Survivin, c-myc and Cyclin D1 at 3 day |
| **Non-canonical Wnt pathway – Experimental findings** | | | | | | | |
| Borsello et al. (2003) | Male ICR/CD1 mice | Transient MCAo  (120 minutes) | D-JNKI-1  (15.7ng/2ul) | Intracerebroventricular | Immediately on onset |  | - JNK activation between 1 and 24 hours after ischemia  D-JNKI-1 :  - Decreased infarct volume  - Had a neuroprotective effect |
| Ferrer et al. (2003) | Male Sprague-Dawley rats | Global cerebral ischemia | N/A | N/A | N/A |  | - JNK and c-Jun phosphorylation is increased very early within the infarct core and peri-lesional region |
| Brecht et al. (2005) | Male JNK-/- mice and JunAA mice | Permanent MCAo | N/A | N/A | N/A |  | JNK-/- mice :  - Decreased infarct volume |
| Rikitake et al. (2005) | Male wildtype | Transient MCAo  (120 minutes) | 1) Fasudil  (1, 3, or 10 mg/kg/day)  2) Y-27632  (10 mg/kg/2day) | Intraperitoneal | Immediately on onset | - Vascular | Fasudil :  - Inhibited ROCK activity  - Increased level of eNOS  - Ameliorated neurological functions  - Decreased infarct volume  - Increased basal and regional CBF only at 10mg/kg  Y-27632 :  - Ameliorated neurological functions  - Decreased infarct volume |
| Repici et al. (2007) | Male P14 Wistar rats | Focal ischemia | D-JNKI-1  (11 mg/kg) | Intraperitoneal | 30 minutes before onset | - Neuronal | - Increased level of c-jun at 3 hours and peaks at 6h in the ischemic core  - Increased level of c-jun at 1 hours and at 6 hours in the penumbra  D-JNKI-1 :  - Inhibited activation of c-jun  - Decreased infarct volume  - Anti-apoptotic effect (CC3) |
| Benakis et al. (2010) | Male ICR/CD1 mice | Transient MCAo  (45 minutes) | D-JNKI1  (0.1mg/kg) | Tail vein | 3 hours after onset | - Inflammatory | - Increased level of c-JunSer63 and ser73 in cortical region as early as 9 hours  - Increased level of c-JunSer63 and ser73 in the cortical and striatal regions at 48 hours  D-JNKI1 :  - Did not reduced the recruitment and activation of microglia at 48 hours (CD11b) |
| Vest et al. (2010) | Male C57BL/6 mice | Transient MCAo  (60 minutes) | tatCN21  (1mg/kg) | Intravenous | 1 hours after onset | - Neuronal | tatCN21 :  - Decreased infarct volume at 24 hours  - Attenuated glutamate-mediated neuronal cell death at 24 hours |
| Wu et al. (2012) | Male Sprague-Dawley rats | Permanent MCAo | Fasudil  (10mg/kg) | Intracerebroventricular | 1 hour before onset | - Neuronal | Fasudil :  - Decreased motor deficit  - Anti-apoptotic effect (TUNEL+) |
| Buga et al. (2014) | Male Sprague-Dawley rats | Transient MCAo  (90 minutes) | N/A | N/A | N/A |  | SMCs was modulated by Lef1 and Wnt4a at day 14.  -Wnt5 increased in the old animal in the peri-lesional cortex. |
| Li et al. (2017) | Male TRAF6-Ko and TRAF6-TG mice | Transient MCAo  (45 minutes) | NSC23766 (10mg/kg) | Intracranial | 1 hour after onset | - Neuronal  - Inflammatory | -TRAF6 induced Rac1 activation  TRAF6 Ko mice :  - Decreased the infarct volume  - Ameliorated neurological functions  - Anti-inflammatory effect (TNFα, IL1β, MCP1)  - Decreased oxidative stress (DHE, 4-HNE, 8-OHdG)  - Decreased neuronal death (CC3, BCL2, BAX)  TRAF6-TG mice  - Had the opposite effects compared to TRAF6 Ko mice  NSC23766 :  - Reversed the deleterious effects of TRAF6 overexpression |
| Hiroi et al. (2018) | ROCK1+/−, ROCK2+/−,  mice | Transient MCAo  (60 minutes) | N/A | N/A | N/A |  | ROCK1+/- mice :  - Did not reduced infarct volume  - Did not reduced neurological deficit  ROCK2+/- mice :  - Did not reduced infarct volume  - Did not reduced neurological deficit  - Increased level of eNOS in the penumbra |
| Lu et al. (2020) | Male C57BL/6 mice | Transient MCAo | PFN1 shRNA lentivirus | Intracerebroventricular | 2 weeks before onset | - Inflammatory | PFN1 shRNA lentivirus :  - Decreased RHOA and ROCK2 activity  - The level of ROCK1 remain unchanged  - Ameliorated neurological functions  - Ameliorated the reperfusion in the ipsilateral hemisphere  - Decreased the mRNA expression and protein secretion of M1-microglia (CD32, iNOS, CD86, IL1β, IL6, TNFα)  - Increased the mRNA expression and protein secretion of M2-microglia (CD206, Arg1, YM1/2, and IL10) |
| Zheng et al. (2020) | Male Sprague–Dawley rats | Transient MCAo  (120 minutes) | JNK‐IN‐8 (20mg/kg) | Intracerebroventricular | Immediately on onset | - Inflammatory | JNK-IN-8 :  - Inhibited activation of JNK and NF‐κB signaling  - Ameliorated the neurological function from 3 to 14 days  - Anti-inflammatory effect (IBA1, TNFα, IL6, IL1β) |

**Table I. Summary of major studies in ischemic stroke.** A table summarizing the findings that have investigated Wnt pathway in ischemic stroke pathobiology and therapy. PTGES; prostaglandin E synthase. MCP1; monocyte chemotactic protein-1. ELAM1; endothelial-leukocyte adhesion molecule 1. DHE; dihydroethidium. 4-HNE; 4-hydroxynonenal. 8-OHdG; 8-hydroxy-2 deoxyguanosine. IBA1; ionized calcium-binding adapter molecule 1. Tuj1; Neuron-specific class III beta-tubulin.
