# Supplementary material for: Wnt Pathway: An Emerging Player in Vascular and Traumatic Mediated Brain Injuries
Source: Front Physiol. 2020 Sep 18;11:565667. doi: 10.3389/fphys.2020.565667 (PMC7530281; doi:10.3389/fphys.2020.565667)
Supplement: Supplementary file 2 [file Table_2.DOCX]

| **Studies** | **Species** | **Stroke type/ Experimental models** | **Treatment** | **Route of delivery** | **Time of delivery** | **Biological processes** | **Main effects**  **(Key markers used)** |
| --- | --- | --- | --- | --- | --- | --- | --- |
| **Canonical and non-canonical Wnt pathway - Clinical findings** | | | | | | | |
| Tran et al. (2016) | Human | Spontaneous non-traumatic intracerebral hemorrhage | N/A | N/A | N/A | - Vascular | - Decreased level of β-catenin and tight junction (claudin-1) |
| Zhao et al. (2011) | Human | Subarachnoid hemorrhage | Fasudil  (30mg/3x/day/14days) | Intravenous | 24 hours after onset | - Vascular | Fasudil :  - Ameliorated clinical’s outcome  - Prevented cerebral vasospasm  - Prevented subsequent ischemic injury |
| Zhao et al. (2006) | Human | Aneurysmal subarachnoid hemorrhage | Fasudil  (30mg/3x/day/14days) | Intravenous | 24 hours after onset | - Vascular | Fasudil :  - Ameliorated clinical’s outcome  - Prevented cerebral vasospasm |
| González-Montelongo et al. (2018) | Human | Spontaneous aneurysmal subarachnoid hemorrhage | N/A | N/A | N/A | - Vascular | RHOA level in peripheral blood mononuclear cells might be related with aSAH severity and cerebral vasospasm |
| Lai et al. (2019) | Human | Intracranial aneurysm | N/A | N/A | N/A |  | - Decreased level of APC |
| **Canonical Wnt pathway – Experimental findings** | | | | | | | |
| Krafft et al. (2012) | Male CD1 mice | 30μl of blood injection | α7nAChR agonist  (12mg/kg) | Intraperitoneal | 1 hour after onset | - Neuronal | α7nAChR agonist :  - Decreased infarct volume at 24 hours and 72 hours  - Ameliorated neurological function at 24 hours and 72 hours  - Anti-apoptotic effect (CC3, TUNEL+) |
| Krafft et al. (2013) | Male CD1 mice | 30μl of blood injection | PHA-543613  (12mg/kg) | Intraperitoneal | 1 hour after onset | - Vascular | PHA-543613 :  - Decreased level of GSK3β at 24 hours  - Decreased brain oedema  -Ameliorated sensitomotor function  - Preserved BBB integrity (Evans blue, claudin-3, claudin-5) |
| Chen et al. (2015) | Male Sprague-Dawley rats | SAH | 1) rNorrin  (50ng/μl)  2) Frizzle 4 siRNA | Intracerebroventricular | 1) Starting 3 hours after onset  2) Starting 48 hours before onset | - Vascular | rNorrin  - Decreased brain oedema  - Preserved BBB integrity (occludin, VE-Cadherin, ZO1)  Frizzle 4 siRNA :  - Inhibited the beneficial effect of rNorrin |
| Zheng et al. (2016) | Male Sprague-Dawley rats | 60μl of blood injection | LiCl  (60mg/kg/2x/days) | Intraperitoneal | 2 hours after onset | - Neuronal  - Inflammatory | LiCl :  - Had a neuroprotective effect  - Anti-inflammatory effect (MPO, OX42)  - Anti-apoptotic effect (TUNEL+) |
| Tran et al. (2016) | Male iCKO mice | N/A | N/A | N/A | N/A | - Neuronal  - Vascular | - Decreased level of β-catenin  - Decreased level of tight junction (claudin-1, claudin-3)  - Caused petechial hemorrhaging (FITC-BSA)  - Anti-inflammatory effect (CD45)  - Anti-apoptotic effect (TUNEL+) |
| Zhao et al. (2017) | Male C57Bl/6 mice | 5μl of Collagenase injection | 6-BIO  (8μg/kg) | Intraperitoneal | - Immediately or 2 hours after onset for acute phase  - 72 hours after onset and every 48 hours for chronic phase | - Neuronal  - Vascular | 6-BIO :  - Decreased hematoma size  - Increased neurogenesis (BDNF, BrdU+/NeuN+)  - Increased angiogenesis (VEGF, BrdU+/GLUT1+)  - Anti-apoptotic effect (BCL2, TUNEL+, TUNEL+/NeuN+) |
| Li et al. (2017) | Male Sprague–Dawley rats | 100μl of blood injection | siDkk1  (10μl) | Intracerebroventricular | 48 hours before onset | - Vascular | - Increased level of Dkk1 at 24 hours and 3 days  siDkk1 :  - Decreased oedema  - Preserved BBB integrity (Evans blue, ZO1) |
| Li et al. (2018) | Male Sprague-Dawley rats | 80μL semi-coagulated autologous whole blood | 1) TWS119  (30 mg/kg)  2) LiCl  (60 mg/kg) | Intraperitoneal | 2 hours after onset | - Vascular | TWS119 :  - Ameliorated sensorimotor function  - Decreased brain oedema  LiCl :  - Increased the p-GSK3β(ser9)/GSK3β ratio  - Decreased the p-β-catenin/β-catenin ratio  - Ameliorated sensorimotor function  - Decreased brain oedema  - Preserved BBB integrity at 24 hours (Evans blue, claudin-1, claudin-3) |
| Liu et al. (2018) | Male Sprague-Dawley rats | 80μl of whole blood | LiCl  (60mg/kg/2x/day) | Intraperitoneal | 2 hours after onset | - Neuronal | LiCl :  - Ameliorated spatial learning  - Decreased memory impairment  - Decreased excitotoxic index level in ipsilateral hippocampus from day 1 to day 14  - Suppressed cell death in CA1, CA3, and DG subregions at day 3 |
| Wang et al. (2019a) | Male Sprague–Dawley rats | 0.5U in 0.5μl  sterile saline of Collagenase injection | 1) Mino  (45mg/kg)  2) Mino  (22.5mg/kg2x/2days)  3) siDkk1  (10μl) | 1) Intraperitoneal  2) Intraperitoneal  3) Intracerebroventricular | 1) Immediately on onset  2) 48 hours before onset | - Vascular  - Inflammatory | Mino :  - Decreased level of Dkk1  - Increased level of Wnt1 and β-catenin  - Decreased brain oedema  - Preserved BBB integrity (Evans blue, occludin)  - Anti-inflammatory effect (TNF, IL6)  siDkk1 :  - Ameliorated result of mino treatment alone |
| Wang et al. (2019b) | Male Sprague-Dawley rats | SAH | RhWnt1  (1ng) | Intracerebroventricular | 6 hours before killed animal | - Neuronal  - Inflammatory | RhWnt1 :  - Decreased brain oedema  - Ameliorated neurological functions  - Anti-inflammatory effect (CD206)  - Anti-apoptotic effect (TUNEL+) |
| Ruan et al. 2(020) | Male Sprague-Dawley rats | SAH | 1) Recombinant wnt3a  (0.4 and 1.2μg/kg)  2) XAV939  (8mg/kg) | Intranasal | 1) 1 hour after onset  2) Immediately on onset | - Neuronal | - Decreased the level of Frz1 from 6 to 72 hours  -Increased level of aldolase C, axin from 6 to 72 hours  Wnt3a :  - Increased level of Wnt3a, Fzd1, aldolase C and β-catenin  - Decreased level of axin  - Ameliorated short-, mid and long-term neurofunctions  - Decreased brain oedema  - Had a neuroprotective effect  - Decreased apoptosis at cellular and subcellular level (Bcl-2/Bax ratio, CC3, TUNEL+)  XAV939 :  - Inhibited the beneficial effect of Wnt3a |
| **Non-canonical Wnt pathway – Experimental findings** | | | | | | | |
| Yatsushige et al. (2005) | Mongrel dogs | SAH | SP600125  (30μmol/l/3days) | CSF in cisterna magna percutaneously |  | - Vascular  - Inflammatory | SP600125 :  - Decreased level of p-JNK/JNK ratio  - Decreased level of p-c-Jun/c-Jun ratio  - Ameliorated appetite, activity and neurological functions  - Attenuated angiographic and morphological vasospasm of the basilar artery  - Anti-inflammatory effect (IL6)  - Extremely limited infiltration of leukocyte (CD4, CD8), neutrophil (MPO) and macrophage (CD68) |
| Yatsushige et al. (2008) | Mongrel dogs | SAH | SP600125  (30μmol/l/3days) | CSF in cisterna magna percutaneously |  | - Neuronal  - Inflammatory | SP600125 :  - Reduced the number of OX42  - Anti-inflammatory effect  - Anti-apoptotic effect (CC3, TUNEL+) |
| Wan et al. (2009) | Male Sprague-Dawley rats | 100μL of autologous blood | DFX  (100mg/kg) | Intraperitoneal | 2 hours after onset |  | DFX :  - Decreased level of p-JNK  - Ameliorated neurological functions from day 1 to day 28 |
| Fujii et al. (2012) | Male Sprague-Dawley rats | SAH | 1) Hydrofasudil  (10mg/kg)  2) Y-27632  (10mg/kg) | Intraperitoneal | 1) 30 minutes or 6 hours after onset  2) 30 minutes after onset | - Vascular | - Increased level of ROCK at 24 hours  Hydrofasudil :  - Ameliorated neurological functions  - Decreased brain oedema  - Preserved BBB integrity (Evans blue, occludin, ZO1)  Y-27632 :  - Ameliorated neurological functions  - Decreased brain oedema |
| Huang et al. (2012) | Male CD1 mice | 30μl of  autologous blood | 1) Y-27632  (20nmol/kg) | Intracerebroventricular | 30 minutes after onset |  | - RAC1 remain unchanged at 72 hours  - Increased level of RHOA at 72 hours  Y-27632 :  - Ameliorated neurological functions  - Decreased brain oedema |
| Naraoka et al. (2013) | Female Japanese white rabbits | SAH | 1) Pitavastatin  (0.8mg/kg/5days)  2) Fasudil (3.0mg/kg/2x/5days) | 1) Oral (gavage)  2) Intravenous | Immediately on onset | - Vascular | Pitavastatin :  – Decreased level of RHOA in the VSMc  - Increased level of eNOS in endothelial cells  Pivastatin and Fasudil co-treatment :  - Decreased level of RHOA in VSMc  - Increased level of eNOS in endothelial cells |
| Edvinsson et al. (2014) | Male Sprague-Dawley rats | SAH | 1) KN93  (0.0501μg/kg/2x/3days) | Cisternal | 1 hour after onset | - Vascular | - Increased level of CAMKII in basilar and middle cerebral arteries at 24 and 72 hours  KN93 :  - Decreased level of CAMKII  - Decreased contractility  - Ameliorated sensorimotor function at 48 hours |
| Zhao et al. (2016) | Male Sprague-Dawley rats | 50μl of  autologous blood  0.2U of collagenase in in 1ul of sterile saline of collagenase | A-438079  (30mg/kg/2days) | Intraperitoneal | 30 minutes or 24 hours after onset | - Neuronal  - Vascular | - P2X7 receptor increased at 3 hours, peaked at 24 hours, returned to normal level at 72 hours  A-438079 :  – Ameliorated neurological functions  - Decreased brain oedema  - Decreased neuronal degeneration  - Preserved BBB integrity (Evans blue, occludin, ZO1, VE-cadherin) |
| Fan et al. (2017) | Male Sprague-Dawley rats | SAH | siEphA4  (5ng/10μl) | Intracerebroventricular | 48 hours before onset | - Neuronal  - Vascular | siEphA4 :  - Ameliorated neurological functions  - Decreased brain oedema  - Preserved BBB integrity (Evans blue, claudin-5, ZO1)  - Anti-apoptotic effect (CC3) |
| Zhou et al. (2018) | Male Sprague–Dawley rats | SAH | DHLA  (10mg/kg) | Intraperitoneal | 1 hour after onset | - Inflammatory | DHLA :  - Decreased level of CAMKII and JNK  - Ameliorated short- and long-term neurobehavior functions  - Decreased lysosomal rupture in microglia (LAMP1)  - Anti-inflammatory effect (IL1β) |
| Akhter et al. (2018) | Male CD1 mice | 0.05U of collagenase in 1μL of saline | 1) Fasudil  (10 mg/kg)  2) KD025  (200mg/kg) | Intraperitoneal | 1. hour after onset |  | Fasudil :  - Did not ameliorated neurological functions  - Did not decreased hematoma size  KD025 :  - Did not ameliorated neurological functions  - Did not decreased hematoma size  -Took longer time to stop bleeding |
| Ling et al. (2019) | Male Sprague-Dawley rats | SAH | siJNK1  (3mg/kg/3days) | Caudal vein | Immediately on onset | - Neuronal | siJNK1 :  - Decreased the level of JNK  - Ameliorated neurorehabilitation  - Ameliorated survival times  - Anti-apoptotic effect (Caspace-3, BCL2, BAX) |
| Xu et al. (2020) | Male CD1 mice | 0.075U of collagenase in 0.5μl of sterile saline | 1) rDkk3 or SRP6268  (0.5, 1.5 or 5μg/10μl)  2) SiKrm1 or siDvl1 (100pmol/10μl) | 1) Intranasal  2) Intracerebroventricular | 1) 1 hour after onset  2) 48 hours before onset | - Inflammatory | rDkk3 :  - Ameliorated neurological functions  - Decreased brain oedema  - Anti-inflammatory effect (IL1β, TNFα)  siKrm1 or siRNA Dvl1 :  Inhibited the beneficial effects of rDkk3 |

**Table II. Summary of major studies in haemorrhagic stroke.** A table summarizing the findings that have investigated Wnt pathway in haemorrhagic stroke pathobiology and therapy. MPO; Myeloperoxidase.
