# Supplementary material for: Wnt Pathway: An Emerging Player in Vascular and Traumatic Mediated Brain Injuries
Source: Front Physiol. 2020 Sep 18;11:565667. doi: 10.3389/fphys.2020.565667 (PMC7530281; doi:10.3389/fphys.2020.565667)
Supplement: Supplementary file 3 [file Table_3.DOCX]

| **Studies** | **Species** | **Stroke type/ Experimental models** | **Treatment** | **Route of delivery** | **Time of delivery** | **Biological processes** | **Main effects**  **(Key markers used)** |
| --- | --- | --- | --- | --- | --- | --- | --- |
| **Canonical and non-canonical Wnt pathway - Clinical findings** | | | | | | | |
| Ke et al. (2020) | Human | Severe TBI | N/A | N/A | N/A |  | - High levels of Dkk1 in serum  - Close association of Dkk1 levels with the increasing severity and rising short‐term mortality of severe TBI |
| Brabeck et al. (2004) | Human | Closed TBI | N/A | N/A | N/A |  | - Increased expression of RHOA+ and RHOB+ cells at the lesion site  - Increased accumulation of RHOA+ cells in the temporal brain from 7 hours to several months  - Increased accumulation of RHOB+ cells in the temporal brain from 12 to 48 hours  - RHOA and RHOB are mainly expressed in astrocyte at 6 months |
| **Canonical Wnt pathway - Experimental findings** | | | | | | | |
| Wu et al. (2008) | Male Wistar rats | CCI model | Simvastatin  (1mg/kg/14days) | Oral (gavage) | 24 hours after onset | - Neuronal  - Vascular | Simvastatin :  - Improved cognitive function  - Enhanced neurogenesis (BrdU+/NeuN+, BDNF)  - Enhanced angiogenesis (VEGF) |
| Zhu et al. (2010) | Male CD1 mice | CCI model | LiCl  (1mmol/kg/days) | Intraperitoneal | Immediately on onset | - Neuronal  - Inflammatory | LiCl :  - Improved spatial learning, memory ability  - Reduced Oedema  - Attenuated neuronal degeneration  - Anti-inflammatory effect (IL1β) |
| Dash et al. (2010) | Male Sprague-Dawley rats | CCI model | Sodium valproate (VPA) (400mg/kg/5days, 100mg/kg/5days)  . | Intraperitoneal | 30 minutes and 3 hours after onset | - Neuronal | VPA :  - Improved motor function and spatial memory  - Decreased hippocampal dendritic damage (MAP2) |
| White et al. (2010) | Male and female BATGAL mice | CCI model | N/A | N/A | N/A | - Neuronal | - BATGAL+ cells were increased at 7 days in the cortex  - BATGAL+ cells were increased from 3 days to 7 days in the subcallosal zone  - β-catenin increased in proliferating NG2 progenitors and astrocytes at day 3 and day 7 |
| Dash et al. (2011) | Male Sprague-Dawley rats | CCI model | 1) LiCl  (25mg/ml/5days)  2) SB-216763 (5mg/kg/5days) | 1) Subcutaneous  2) Intraperitoneal | 30 minutes after onset | - Neuronal | LiCl :  - Inhibited GSK3ß  - Reduced hippocampal cell loss (NeuN+)  - Improved learning and memory  SB-216763 :  - Increased level of GSK3β  - Decreased level of p-LRP6  - Improved motor conditions  - Exerted no neuroprotection |
| Zhao et al. (2012) | Male Sprague-Dawley rats | CCI model | N/A | N/A | N/A |  | -Increased level of p-GSK3β-ser9 from 24 to 72 hours in the adjacent cortex  - Increased level of p-β-catenin at 4 hours in the adjacent cortex  -p-GSK3β-ser9 and p-β-catenin were mainly found in neurons at 4 hours in the border zone region adjacent to the cortical lesion |
| Yu et al. (2012a) | Male C57Bl/6 mice | CCI model | LiCl  (1.5mEq/kg/14days) | Intraperitoneal | 15 minutes after onset | - Neuronal  - Vascular  - Inflammatory | LiCl :  - Decreased volume lesion at day 3  - Preserved BBB integrity at day 3 (Evans Blue, MMP9)  - Anti-inflammatory effect at day 3 (COX2+, F4/80+)  - Reduced neuronal degeneration at day 3  -Ameliorated motor coordination, hyper-locomotor activity and anxiety-like behaviors at day 7, 14 and 21 |
| Yu et al. (2012b) | Male C57Bl/6 mice | CCI model | LiCl  (1.5mEq/kg/days) | Intraperitoneal | 15 minutes after onset |  | LiCl :  - Decreased Aβ and Tau pathology at day 3  - Ameliorated cognitive function at weeks 3 |
| Wu et al. (2012) | Wistar rats | CCI model | Simvastatin  (1mg/kg/14days) | Oral | 24 hours after onset | - Neuronal | Simvastatin :  - Ameliorated neurological functional recovery  - Reduced axonal injury (Synaptophysin, Neurofilament H)  - Enhanced neurite outgrowth (Tuj1+) |
| Wu et al. (2013) | Male Sprague-Dawley Rats | Weight drop model | N/A | N/A | N/A |  | -Increased level of p-GSK3β-ser9 from day 1 to day 14 in the cortex  -Increased level of p-β-cetenin from day 1 to day 7 in the cortex  - Correlation between SGK expression with p-GSK3β-ser9 and β-catenin |
| Yu et al. (2013) | Male C57BL/6 mice | CCI model | 1) LiCl  (1mEq/kg/days)  2) Valproate  (200mg/kg) | Intraperitoneal | 15 minutes after onset | - Neuronal  - Vascular | LiCl :  - Ameliorated long-term functional recovery  LiCl and Valproate cotreatment:  - Attenuated brain lesion  - Ameliorated long-term functional recovery  - Decreased neuronal degeneration  - Preserved BBB integrity (IgG) |
| Zou et al. (2013) | Male Sprague-Dawley rats | CCI model | N/A | N/A | N/A |  | - CtBP2 associated with astrocyte proliferation via the canonical Wnt pathway |
| Zhang et al. (2013) | Male C57BL/6 mice | LFP model | N/A | N/A | N/A | - Neuronal | - Survivin increased in DCX+ immature neurons in the DG of the hippocampus |
| Zhang et al. (2016) | Male Sprague-Dawley rats | CCI model | Acupuncture |  | Immediately on onset |  | Acupuncture :  - Increased level of Wnt3a, β-catenin from day 7 to day 14 in the cortex.  - Increased level of SOX2 from day 3 to day 7 |
| Zhao et al. (2016) | Male C57BL/6 mice | CCI model | 1) IV-MSCs  (10^6^ cells)  2) Recombinant Wnt3a (400ng) | Tail vein | 2 hours and 24 hours after onset | - Neuronal | IV-MSCs :  - Increased level of Wnt3a and active β-catenin in hippocampus  - Increased level of Wnt3a in serum  - Increased neurogenesis in dental gyrus (DCX+)  Wnt3a :  - Ameliorated neurological function  - Provided neuroprotection  - Increased neurogenesis (DCX+) |
| Wang et al. (2016) | Male C57BL/6 mice | CCI model | 1) bFGF  (0.5μg/g)  2) LY294002  (50 nmol/kg) | 1) Intranasal  2) Left striatum | 1) 1 hour before onset | - Vascular | bFGF :  - Increased level of β-catenin and p120-catenin  - Increased level of GTP-RAC1/total RAC1 ratio  - Decreased level of GTP-RHOA/total RHOA ratio  - Preserved BBB integrity (Evans blue, FITC-dextran permeability)  LY294002 :  - Inhibited the beneficial effect of bFGF |
| Lu et al. (2017) | Male Sprague-Dawley rats | CCI model | N/A | N/A | N/A |  | - Increased level of Dixdc1 in ipsilateral cortex and linked to astrocyte proliferation |
| Salehi et al. (2018) | Male C57BL/6 and TCF:LEF1:H2B-GFP mice | CCI model | N/A | N/A | N/A | - Vascular | - Decreased level of cytoplasmic and nuclear β-catenin from day 3 to day7  - Increased level of cyclin D1 from day 1 to day7  - Increased level of Wnt5a at day 7  - Increased level of β-catenin from day1 to day 7 in vessels  - Increased Wnt-GFP+ cells within vessels at day 1  -Increased fraction of Wnt-GFP+ vessels segment at day 7 |
| Zhang et al. (2018) | Male C57BL/6 mice | CCI model | Wnt3a  (2ug/kg/7days) | Intranasal | 1 hour after onset | - Neuronal  - Vascular  - Autophagy | Wnt3a :  - Increased level of Wnt3a and β-catenin  - Decreased sensiromotor dysfunction  - Had a regenerative and neurogenerative effect (BrdU+/NeuN+, BrdU+/GLUT1+)  - Increased angiogenesis (VEGF)  - Preserved BBB integrity (MMP9)  - Anti-apoptotic effect (CC3, BCL2)  - Anti-autophagic effect (LC3+/NeuN+, LC3-II, Beclin-1) |
| Zhu et al. (2018) | Male Sprague-Dawley rats | FPI model | Docosahexaenoic acid  (370mg/kg or 740mg/kg) | Intragastric | 30 minutes after onset | - Neuronal | Docosahexaenoic acid :  - Anti-apoptotic effect (BCL2, BAX, CC3) |
| He et al. (2019) | Male C57BL/6 mice | Weight drop model | Hyperbaric oxygen | N/A | 1 hour after onset | - Neuronal | Hyperbaric oxygen :  - Anti-apoptotic effect in the perilesional cortex (TUNEL+) |
| Krishna et al. (2019) | Male Sprague Dawley rats | FPI model | Blueberry Supplementation  (5% w/w/14days) | Oral | Immediately on onset |  | Blueberry Supplementation :  - Mitigated effect on spatial learning and memory performances  - Decreased anxiety |
| Chang et al. (2020) | Male Sprague-Dawley rats | CCI model | Wnt3a  (50ng/4days) | Intranasal | Immediately on onset | - Neuronal | Wnt3a :  - Ameliorated behavior and motor function  - Ameliorated regeneration in the injured cortical neurons (NeuN+, GFAP+) |
| Wei et al. (2020) | Male Sprague-Dawley rats | Weight drop model | Thyroxine  (6.5, 10 or 15.4μg/kg) | Intragastric | 6 hours after onset | - Neuronal  - Inflammatory | Thyroxine :  - Increased level of Wnt3a, β-catenin and Notch1 at 7 day  - Ameliorated regeneration (GFAP+, S100β+)  - Anti-apoptotic effect (BCL2, BAX) |
| **Non-canonical Wnt pathway – Experimental findings** | | | | | | | |
| Atkins et al. (2006) | Male Sprague-Dawley rats | CCI model | N/A | N/A | N/A | - Neuronal | - CAMKII activated in DG, CA1, CA3 and cortex at 30 minutes |
| Dubreuil et al. (2006) | Male Sprague–Dawley rats | FPI model | N/A | N/A | N/A |  | - RHOA activation was observed from 24 hours to 3 days in the cortex and at 3 days in the ipsilateral hippocampus |
| Folkerts et al. (2007) | Male Sprague-Dawley rats | LFP model | N/A | N/A | N/A |  | - Increased p-CAMKII activity in the ipsilateral brain from 10 minutes to 24 hours  - p-CAMKII expression was increased in the overlaying cortex and hippocampus at 30 minutes  - p-CAMKII was absent of CA3 neurons |
| Niu et al. (2012) | Male Sprague–Dawley rats | CCI model | Stealth RNAi plus Invivofectamine mixture  (5μl of 1 mg/ml) | Hippocampus | 48 hours before onset |  | - Increased level of Frd2 and Wnt5a  - Increased level of Fzd2, Wnt5a and p-CAMKII in hippocampus  - Increased intracellular Ca2+ in injured hippocampal cells  Stealth RNAi plus Invivofectamine mixture :  - Reduced level of Frd2, Wnt5a, p-CAMKII  - Reduced intracellular Ca2+ in injured hippocampal cells |
| Mao et al. (2016) | Male Sprague-Dawley rats | CCI model | Saikosaponin  (5, 10 and 20mg/kg/3days) | Intravenous | 15 minutes after onset | - Vascular  - Inflammatory | Saikosaponin :  - Decreased the neurological dysfunction  - Decreased oedema  - Preserved BBB integrity (Evans Blue, MMP9, occludin, AQP4)  - Anti-inflammatory effect (IL6, TNFα) |
| Bye et al. (2016) | Male C57BL/6 mice | CCI model | Y-27632  (20μM, 3μg/kg/1week)  Y-27632  (90μM, 3μg/kg/4weeks) | Intracerebroventricular | Immediately on onset | - Neuronal  - Inflammatory | Y-27632 :  - Decreased motor dysfunction  - Had only a little effect on neurogenesis at day 35 (BrdU+/NeuN+)  - Did not affected neuroinflammation at day 35 (CD11b, GFAP) |
| Mulherkar et al. (2017) | Male RHOA CKO mice | CCI model | N/A | N/A | N/A |  | - Preserved motor and cognitive function at day 14  - Did not influencing the contusion volume at day 14 |
| Pan et al. (2018) | rats | CCI model | N/A | N/A | N/A |  | - CAMKIIδ expression increased around the injured area and peaked at day 3 |
| Rehman et al. (2018) | Male C57BL/6 mice | Repetitive CCI model | SP600125  (10 or 20mg/kg/days) | Intraperitoneal | Immediately on onset | - Neuronal  - Vascular  - Inflammatory | SP600125 :  - Ameliorated motor function and behavioral outcomes  - Decreased synaptic loss (Synaptophysin, SNAP23, SNAP25)  - Preserved BBB integrity (Evans blue, claudin-5, ZO1)  - Anti-inflammatory effect (IL1β, TNFα, iNOS)  - Decreased apoptotic neurodegeneration (CC3, BCL2, BAX, PARP1)  - Ameliorated amyloidogenic APP processing |
| Bhowmick et al. (2019) | Male Sprague-Dawley rats | FPI model | N/A | N/A | N/A |  | - Increased level of p-JNK from 12 to 48 hours  - Increased level of p-Erk1/2 from 12 to 48 hours |
| Zhu et al. (2019) | Male C57/BL6 mice | CCI model | Docosahexaenoic acid  (200 mg/kg/2x/3days) | Oral | Immediately on onset | - Neuronal | Docosahexaenoic acid :  - Ameliorated motor function  - Decreased memory dysfunction  - Rescued hippocampal LTP deficits (LTP was induced by 1s of 100Hz tetanus stimuli) |

**Table III. Summary of major studies in traumatic brain injury.** A table summarizing the findings that have investigated Wnt pathway in traumatic brain injury (TBI) pathobiology and therapy. CCI; Controlled Cortical Impact. LFP; Lateral Fluid Percussion. FPI; Fluid Percussion Injury. MAP2; Microtubule-associated protein 2. AQP4; Aquaporin-4. SNAP; Synaptosomal-associated protein. PARP1; poly(ADP-ribose) polymérase 1.
